# Supplementary material for: Unexpected worker mating and colony-founding in a superorganism
Source: Nat Commun. 2023 Sep 7;14:5499. doi: 10.1038/s41467-023-41198-6 (PMC10484907; doi:10.1038/s41467-023-41198-6)
Supplement: Supplementary file 1 — Supplementary Information [file 41467_2023_41198_MOESM1_ESM.pdf]

Supplementary Information:

Title: Unexpected worker mating and colony-founding in a superorganism.

Authors: Mingsheng Zhuang, Thomas J Colgan, Yulong Guo, Zhengyi Zhang, Fugang Liu, Zhongyan Xia, Xueyan Dai, Zhihao Zhang, Yuanjian Li, Liuhao Wang, Jin Xu, Yueqin Guo, Yingping Qu, Jun Yao, Huipeng Yang, Fan Yang, Xiaoying Li, Jun Guo, Mark JF Brown, and Jilian Li.

Supplementary Information: 1

Supplementary Notes 2

A) Queen-worker caste dimorphism in *B. ignitus*, *B. lantschouensis*, *B. montivagus*, and *B. terrestris* 2

B) Transcriptomic analyses of reproductive tissues (spermatheca, vagina, and median oviduct) of inseminated and non-inseminated (control) bumble bee castes . 2

C) Transcriptomic analyses of the brains of inseminated and non-inseminated (control) bumble bee castes 3

D) Transcriptomic analyses of the fat body of inseminated and non-inseminated (control) bumble bee castes 5

E) Transcriptomic analyses of the ovaries of inseminated and non-inseminated (control) bumble bee castes 6

Supplementary Figures 8

Supplementary Notes

A) Queen-worker caste dimorphism in *B. ignitus*, *B. lantschouensis*, *B. montivagus*, and *B. terrestris*

Accurate identification of gynes and workers was essential to enable sampling for our experimental work. Both *B. ignitus* and *B. lantschouensis* belong to the subgenus *Bombus sensu stricto*, which is broadly acknowledged to contain species with distinct queen and worker castes. However, to our knowledge this has not been documented. *B. montivagus* belongs to the subgenus *Megabombus*, a clade less well-known for queen-worker dimorphism. To demonstrate the degree of queen-worker dimorphism in these species we weighed all the females produced by 15 colonies each of *B. ignitus* and *B. lantschouensis*, and from one colony of *B. montivagus* which was dug up in the field and kept in the laboratory. All three species showed distinct queen-worker dimorphism, with a clear gap between castes in terms of weight (Supplementary Figure 1). Consequently, we are confident that we were able to select bees accurately for our experimental work.

B) Transcriptomic analyses of reproductive tissues (spermatheca, vagina, and median oviduct) of inseminated and non-inseminated (control) bumble bee castes

Raw FASTQ files for 40 reproductive tissue (spermatheca, vagina and median oviduct) samples (n = 20 workers; n = 20 queens: Supplementary Data 8) were filtered for quality using fastp[1] (v.0.23.0) to remove adaptors and low-quality sequences. We next aligned filtered sequences from each sample against the latest *B. terrestris* reference genome assembly (obtained from Ensembl Metazoa; release 53, GCA_000214255.1) using the short read aligner, STAR[2] (v.2.7.10a). Mean mapping rate was high (99% of reads mapped per samples) and using gene-level read counts calculated by STAR, transcript expression was detected for 94% of all known genes (n = 11,284/12,008) across all samples. Gene-level counts were loaded into DESeq2[3] (v.1.26.0) to create a DESeq2 object. We provide these raw gene-level counts to aid independent reanalysis (Supplementary Data 8). We next filtered out genes with low read counts across samples before we generated variance-stabilization transformed (VST) counts. Using these counts, we performed hierarchical clustering based on Euclidean distances finding separation of individual based on caste, with the exception for one worker that clustered with queens (Supplementary Figure 10). We removed one aberrant queen sample and performed a principal component analysis (PCA), which also separated samples based on caste: principal component (PC) 1 explained 73% of the variance in the dataset, largely separating workers and queens into two clusters

(Supplementary Figure 11). The second principal component (PC2) separated individuals based on insemination status, explaining a smaller proportion of the variance in the dataset (13%, Supplementary Figure 11).

Using VST counts from DESeq2, we performed a weighted co-expression network analysis for samples from reproductive tissues using WGCNA. Genes clustered into six modules, which ranged in gene assignment from 79 genes ("pink" module) to 9,392 genes ("turquoise" module). We examined correlations between each module and insemination status (treatment), caste, and age (stage), respectively, finding two modules ("red", n = 154 genes; "yellow", n = 840 genes) that had strong positive correlations with treatment ("red", R = 0.67, p = 3e-06; "yellow", R = 0.94, p = 2e-18; Supplementary Figure 12a). The "yellow" module was also positively correlated (R = 0.45, p = 0.005) with age but the significance of the correlation was not as strong as for treatment. For both "red" and "yellow" modules, we identified the hub (driver) genes as a ribosome biogenesis protein BOP1 homologue (LOC100642762), a gene required for ribosomal maturation, essential for translation, and a double-stranded RNA-specific editase Adar (LO100651176), a gene associated with behavior, reproduction, and glucose metabolism in the fruit fly, respectively. Independent Gene Ontology enrichment analyses was performed for genes assigned to each module identifying enrichment of 13 and 54 enriched GO terms for the "red" and "yellow" modules, respectively. For the "yellow" module, enriched terms included 32 BP terms, identifying a diverse range of processes influenced by insemination, including enriched GO terms associated with translation, neurology, immunity, and development (Supplementary Figure 12b).

C) Transcriptomic analyses of the brains of inseminated and non-inseminated (control) bumble bee castes

To determine genes expressed in the brain, an important regulator of behavior, nervous system function, and reproduction, we performed RNA-seq analysis for 72 brain-derived libraries consisting of 48 worker and 24 queen samples, respectively. For the generation of RNA-seq libraries, brains were collected and pooled for bees at three stages of ovarian development and from one of four treatment groups: insemination group (AI Group 1), insemination with diluent (AI Group 2), injection only (AI Group 3), and control (non-inseminated) bees (Supplementary Data 8). For the worker samples, there was four pooled samples per ovarian development stage for each of the four treatments while for the queens, only insemination group (AI Group 1) and control bees were collected. The mean library size for the raw brain samples was 23.9 million PE reads (min: 20.8 million PE reads; max: 25.4 million PE reads) with a mean alignment rate of 99.9% against the reference genome assembly. Of the genes detected as expressed in the brain, 78% of genes (n = 9,103/11,549)

were expressed in all samples (Supplementary Data 8). Similar to as outlined above, we created a DESeq2 object using all brain samples and performed variance-stabilization transformation on counts. Using these counts, we assessed outliers through a hierarchical clustering-based analysis identifying three samples with aberrant expression profiles (Supplementary Figure 13), which were removed from further analysis. A subsequent PCA separated individuals based on stage of ovarian development (PC1: 31% of variance explained) while PC2, which explained 18% of the variance, largely separated individuals based on caste (Supplementary Figure 14).

Given the differences in number of treatments and associated samples per caste, for differential gene expression analysis we analyzed the datasets of each caste independently. For both caste-specific analyses, we used a likelihood ratio test (LRT) that compared a full model consisting of stage of ovarian development ("stage") and treatment with a reduced model consisting of stage only. For the workers, we additionally compared the $\log_2$ fold change values for each treatment group against the control samples applying a significant threshold of FDR < 0.05 and an absolute $\log_2$FC greater than or equal to one ($|\log_2FC| >= 1$) to determine genes that were significantly differentially expressed between a particular treatment group and the control group. Using these criteria, we found that the artificial insemination treatment (AI Group 1), which included bees that received a transfer of semen, had the greatest transcriptional changes compared to control bees with 224 genes differentially expressed, of which 211 had elevated expression in response to treatment (Supplementary Data 8). We found that the other insemination treatments also, in general, resulted in elevated gene expression when compared to the control group but the overall number of differentially expressed genes was lower (AI Group 2 = 80 elevated genes / 86 total genes; AI Group 3 = 63 / 85) than the artificial insemination group. A comparison of differentially expressed genes across the three treatment groups identified 40 DEGs with consistently elevated expression in all three treatments suggestive of a conserved response to the insemination procedure (Supplementary Figure 15). A further 64 DEGs were found in two treatment groups while 123 DEGs were unique to insemination group (AI Group 1, Supplementary Figure 16a), which were enriched for GO terms associated with development, metamorphosis, and transcription (Supplementary Figure 16b).

For the queen samples, which consisted of two treatment groups (insemination group (AI Group 1) and control), we found 59 DEGs (FDR < 0.05; $|\log_2FC| >= 1$) with the majority (n = 52) having elevated expression in response to insemination (Supplementary Data 8). Of this number, 25 genes were also elevated in response to the insemination treatment in workers while one gene was reduced in both castes highlighting certain conserved responses in the brain to insemination across castes.

D) Transcriptomic analyses of the fat body of inseminated and non-inseminated (control) bumble bee castes

To investigate how insemination affects genes expressed in the fat bodies, an important producer of proteins involved in reproduction and egg development, we performed RNA-seq analysis for 72 fat body-derived libraries consisting of 48 worker and 24 queen samples. The mean library size for the raw fat body samples was 24.2 million PE reads (min: 22.3 million PE reads; max: 25.5 million PE reads) with a mean alignment rate similar to the brain samples of 99.9% against the reference genome assembly. Of the genes detected as expressed in the fat bodies, 75% of genes (n = 8,603/11,440) were expressed in all samples (Supplementary Data 8).

Initial clustering-based analysis based on Euclidean distance between samples and principal component analysis revealed three outlier samples, which were removed from downstream analyses. Hierarchical clustering using the reduced sample dataset revealed clustering of samples based on both caste but also stage of ovarian development (Supplementary Figure 17). A similar pattern was also observed with a principal component analysis, whereby the first PC, which explained 40% of the variance in the dataset, separated samples based on stage of ovarian development, while the second PC separated individuals based on caste (Supplementary Figure 18).

Similar to the brain samples, we analyzed each caste dataset independently. For the workers, we compared each treatment group against the control samples finding the artificial insemination treatment (AI Group 1), which included the transfer of semen, induced the greatest transcriptional changes with 208 genes differentially expressed of which 191 had elevated expression in response to treatment (Supplementary Data 8). The diluent treatment (AI Group 2) elicited a similar response in terms of number of DEGs although the proportion of genes with elevated expression (n = 89/114) was reduced compared to AI Group 1. The last treatment group (AI Group 3), which involved bees that were exposed to the injection without liquid elicited the largest response with 185 DEGs in response to treatment with a similar number of genes having elevated (n = 161) or reduced (n = 24) expression in comparison to control samples. Across all treatments, 39 genes were consistently differentially expressed (Supplementary Figure 19). The most unique DEGs were found for the insemination (AI Group 1; n = 108), followed by the injection (AI Group 3) and diluent (AI Group 2) groups, respectively (Supplementary Figure 19 and 20).

For the queens, differential expression analysis between inseminated and control bees identified a total of 125 DEGs (FDR < 0.05; |log2FC| >= 1). In contrast to the brains where

inseminated induced elevated gene expression, the directional change in expression was less strong with approximately a third of DEGs (n = 45) having elevated expression in the fat bodies of inseminated bees with the majority (n = 80) displaying reduced expression compared to control bees. There was only a small number of DEGs that overlapped between the castes with three DEGs having elevated expression in response to artificial insemination in both castes while four genes had reduced expression.

E) Transcriptomic analyses of the ovaries of inseminated and non-inseminated (control) bumble bee castes

To determine the influence of insemination on ovarian gene expression, we performed RNA-seq analysis for 72 ovary-derived libraries consisting of 48 worker and 24 queen samples. The mean library size for the raw ovary samples was 24 million PE reads (min: 20.9 million PE reads; max: 25.5 million PE reads) with a mean alignment rate similar to the other tissues of 99.9% against the reference genome assembly. Of the genes detected as expressed in the ovaries, 81.8% of genes (n = 9487/11602) were expressed in all samples (Supplementary Data 8).

Initial clustering-based analyses of VST gene-level counts using hierarchical clustering and PCA revealed the presence of two outlier samples, which were removed from downstream analyses. Using the filtered dataset, we found samples primarily clustered based on stage of ovarian development (Supplementary Figure 21 and 22). We performed differential expression analysis identifying 156 genes that differed in expression between inseminated (AI Group 1) and control bees, of which, the majority (n = 143 genes) had elevated expression. Insemination with diluent only (AI Group 2) resulted in the significant differential expression of 63 genes with approximately two-thirds (n = 42) having elevated expression in comparison to control. The last group comparison (AI Group 3) identified that injection alone affected the expression of 41 genes of which 28 had elevated expression compared to control. Across all three treatments, 14 genes were consistently differentially expressed in comparison to the control samples (Supplementary Figure 23). We identified a total of 111 genes uniquely differentially expressed in inseminated workers compared to control (Supplementary Figures 23 and 24). These genes were significantly enriched (Fisher's exact test; $P < 0.05$) for GO terms (n = 7 terms) associated with transcription factor activity, neuron projection, and signal transduction (Supplementary Figure 24).

For the queens, we identified 51 genes that differed significantly in expression in response to insemination (LRT, FDR < 0.05; |log2FC| >=1) with the majority (n = 40) have reduced

expression. Three DEGs had conserved elevated expression in inseminated workers compared to the control treatment while two DEGs had conserved reduced expression.

Supplementary Figures

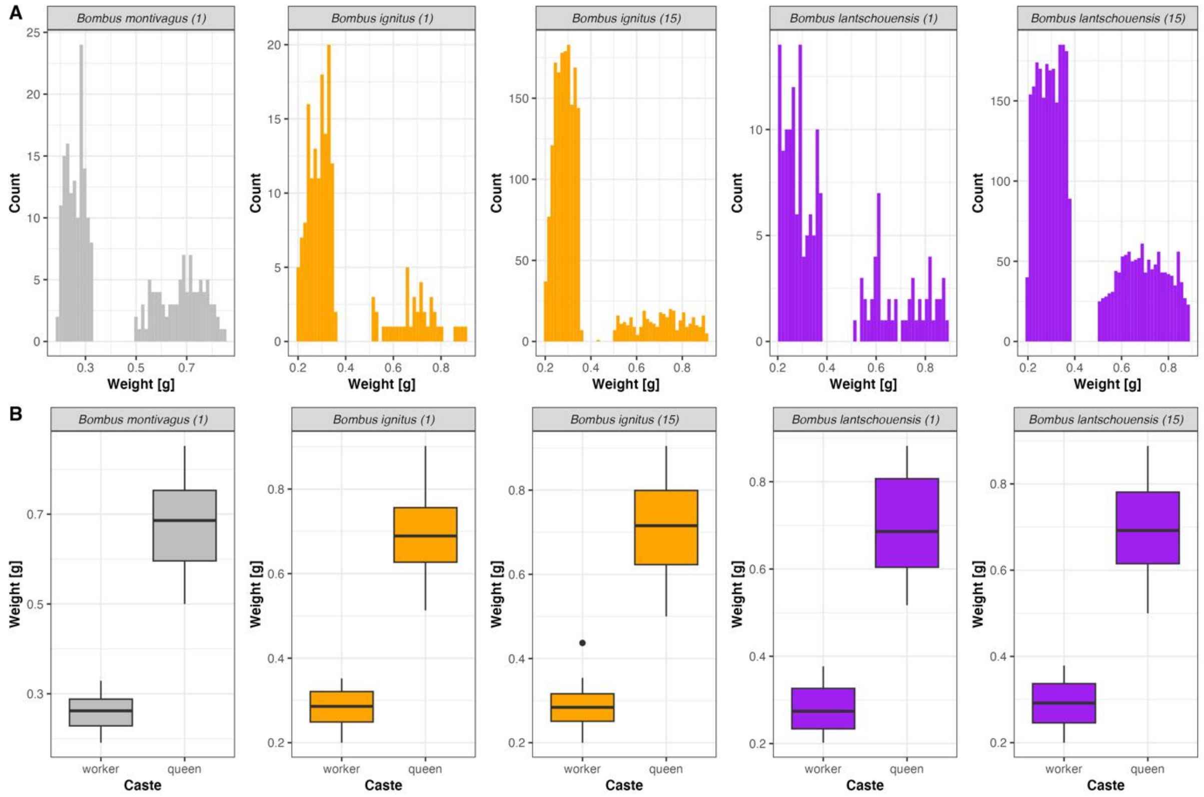

Supplementary Figure 1. Size dimorphism in bumble bee castes. a Histograms displaying weight distributions, a proxy for body size, for all female bees sampled from colonies of three bumble bee species; and **b** Boxplots displaying weight measurements for workers and queens sampled from colonies of three bumble bee species. For each type of plot, species are individually coloured: *Bombus montivagus* = gray, *B. ignitus* = orange, and *B. lantschouensis* = purple with the species name also found in the grid header. For each species, we plot the dataset for a single representative colony indicated by the number found inside the brackets within the grid header while for two of the species, we plot a cumulative plot of all samples collected from all colonies for that species (n = 15). Box plots consist of the box denoting the interquartile range (IQR), bound by the 25th and 75th percentiles, the median line shown within the box, and the whiskers representing the rest of the data distribution with outliers denoted by points greater than ±1.5 x IQR.

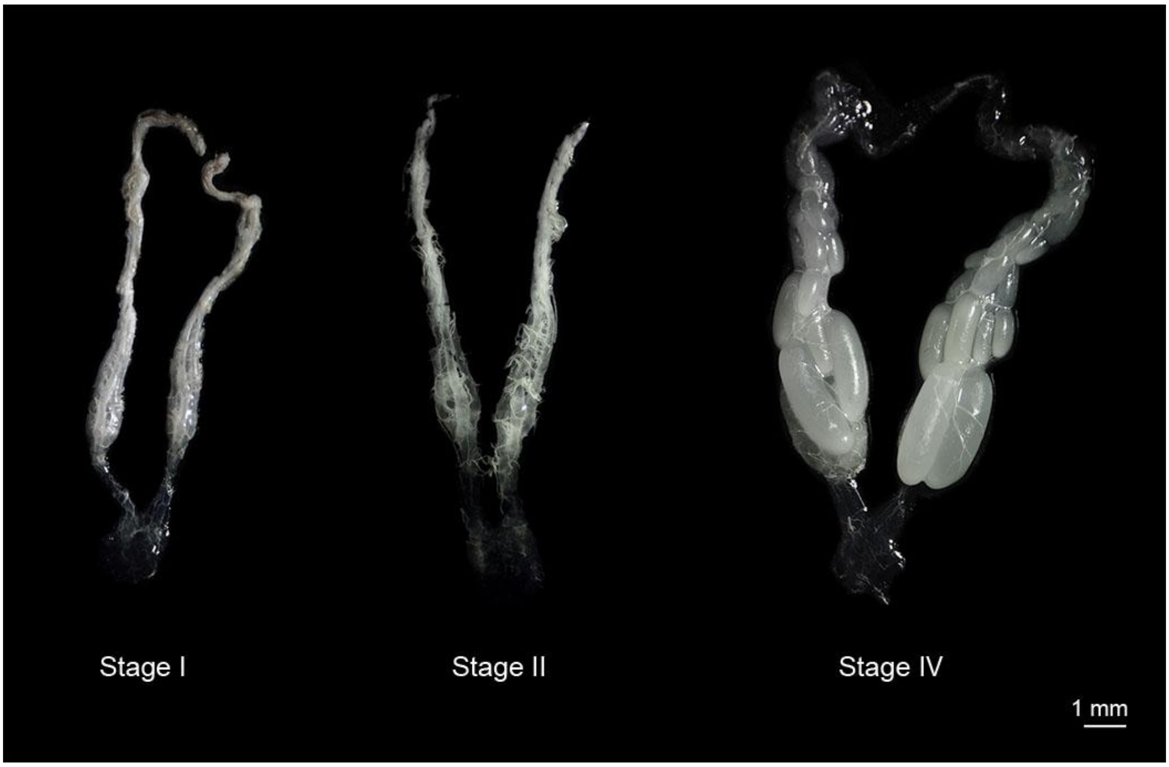

Supplementary Figure 2. Stages of bumble bee ovarian development. In the present study, we characterized ovarian development of reproductive *B. terrestris* workers and queens as belonging to the following stages: stage I, immature ovary with a thread-like appearance; stage II, presence of nutritive cells (i.e., nurse cells) larger than egg cells; and stage IV, the presence of mature eggs (after oviposition). Ovaries were photographed using a camera (Canon. EOS 650D) under a light microscope with 15x magnification. Ten workers and ten queens were dissected for each development stage, with workers and queens having similar ovary development stages. The figure shows three development stages of queen ovaries.

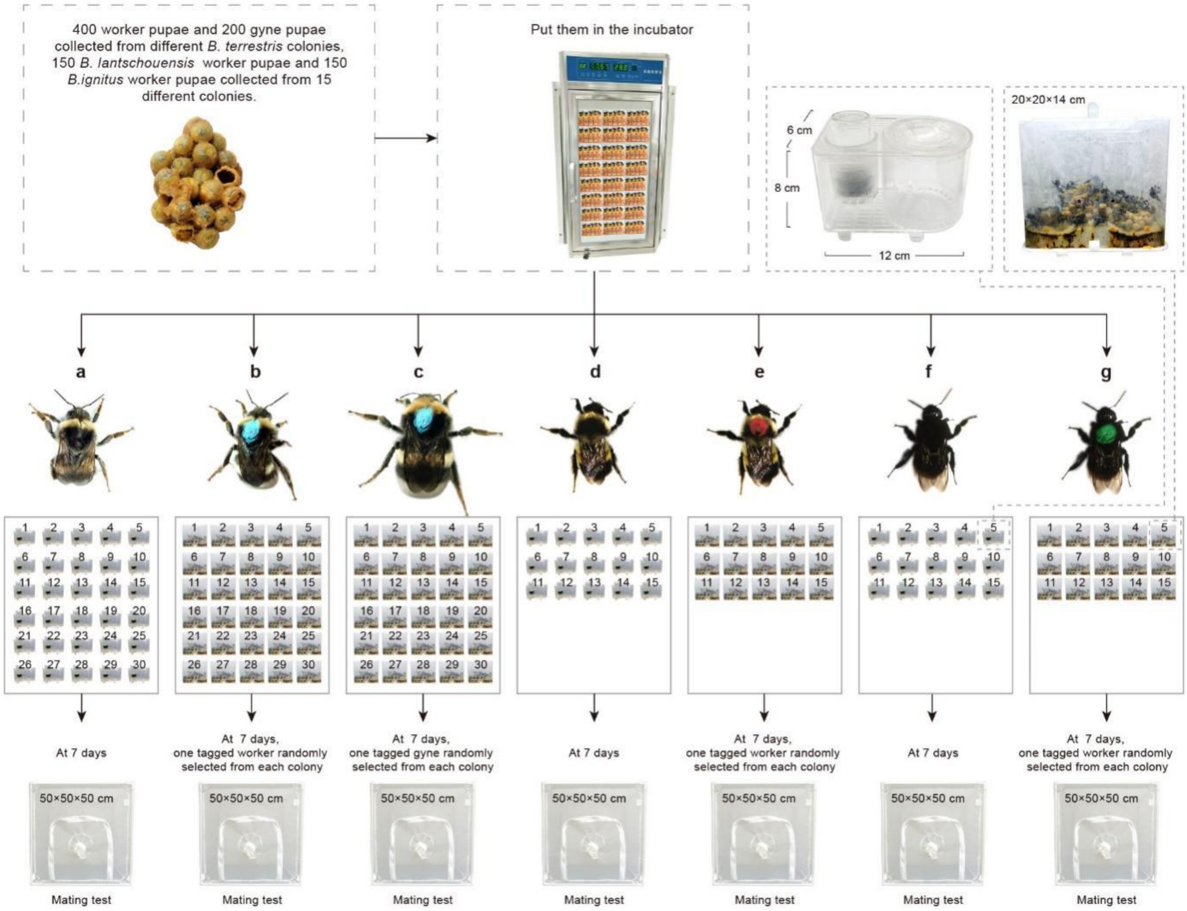

Supplementary Figure 3. The effects of social constraints on mating success in bumble bee workers. Pupae for both workers and queens were collected and incubated to assist in development and monitored daily to track eclosion. Newly eclosed callow bees of each species were entered into the following treatments: **a** after eclosion, each *B. terrestris* callow worker was kept in an individual small plastic box under solitary conditions; **b** after eclosion, each of three *B. terrestris* callow workers were tagged and placed in a queen-right colony; **c** after eclosion, each of three *B. terrestris* gynes were tagged and placed in a queen-right colony; **d** after eclosion, each *B. lantschouensis* callow worker was kept in an individual small plastic box under solitary conditions; **e** after eclosion, each of two *B. lantschouensis* callow workers were tagged and placed in a queen-right colony; **f** after eclosion, each *B. ignitus* callow worker was kept in an individual small plastic box under solitary conditions; **g** after eclosion, each of two *B. ignitus* callow workers were tagged and placed in a queen-right colony. After seven days, the isolated workers, and one tagged worker and one tagged gyne randomly selected from each colony, were given the opportunity to mate with males.

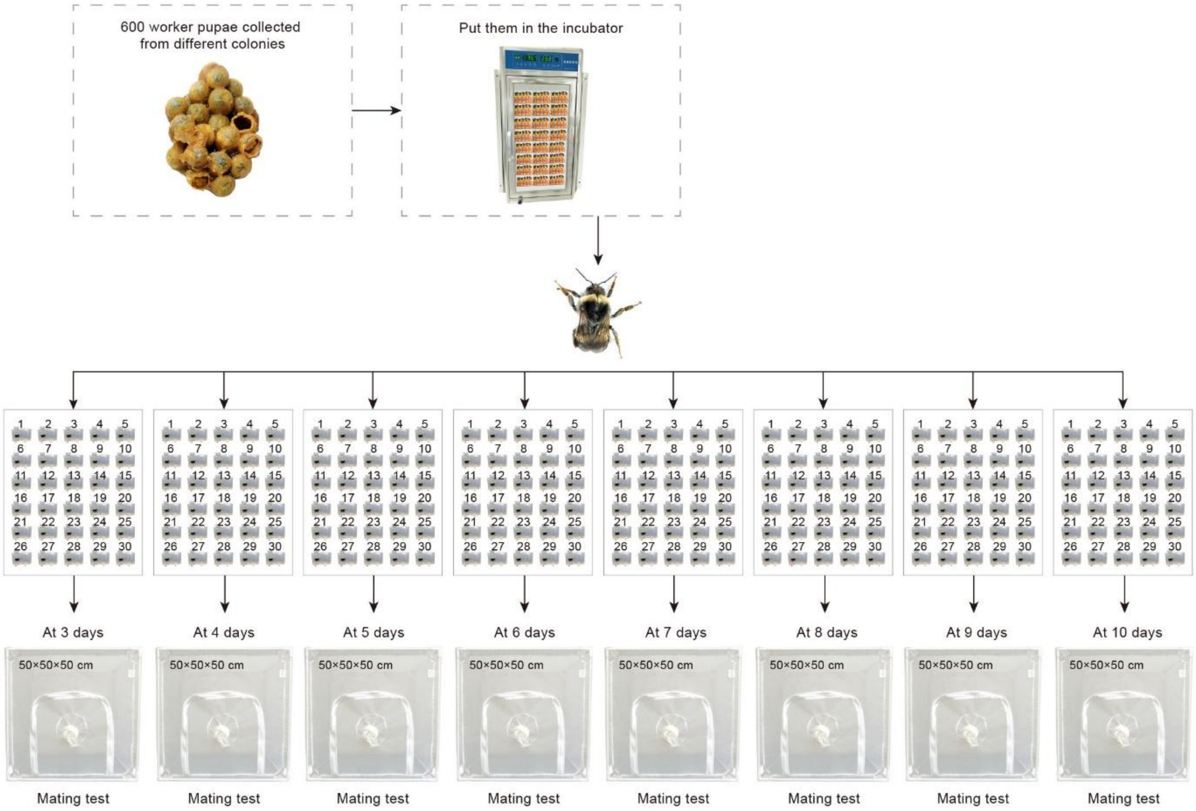

Supplementary Figure 4. Worker age influences mating success. To determine how age may influence mating, we first collected *B. terrestris* worker pupae and placed them into individual boxes before incubating to stimulate development and daily monitoring to track eclosion. We then randomly chose callow workers to be given the opportunity to mate at days 3, 4, 5, 6, 7, 8, 9, and 10 post-eclosion.

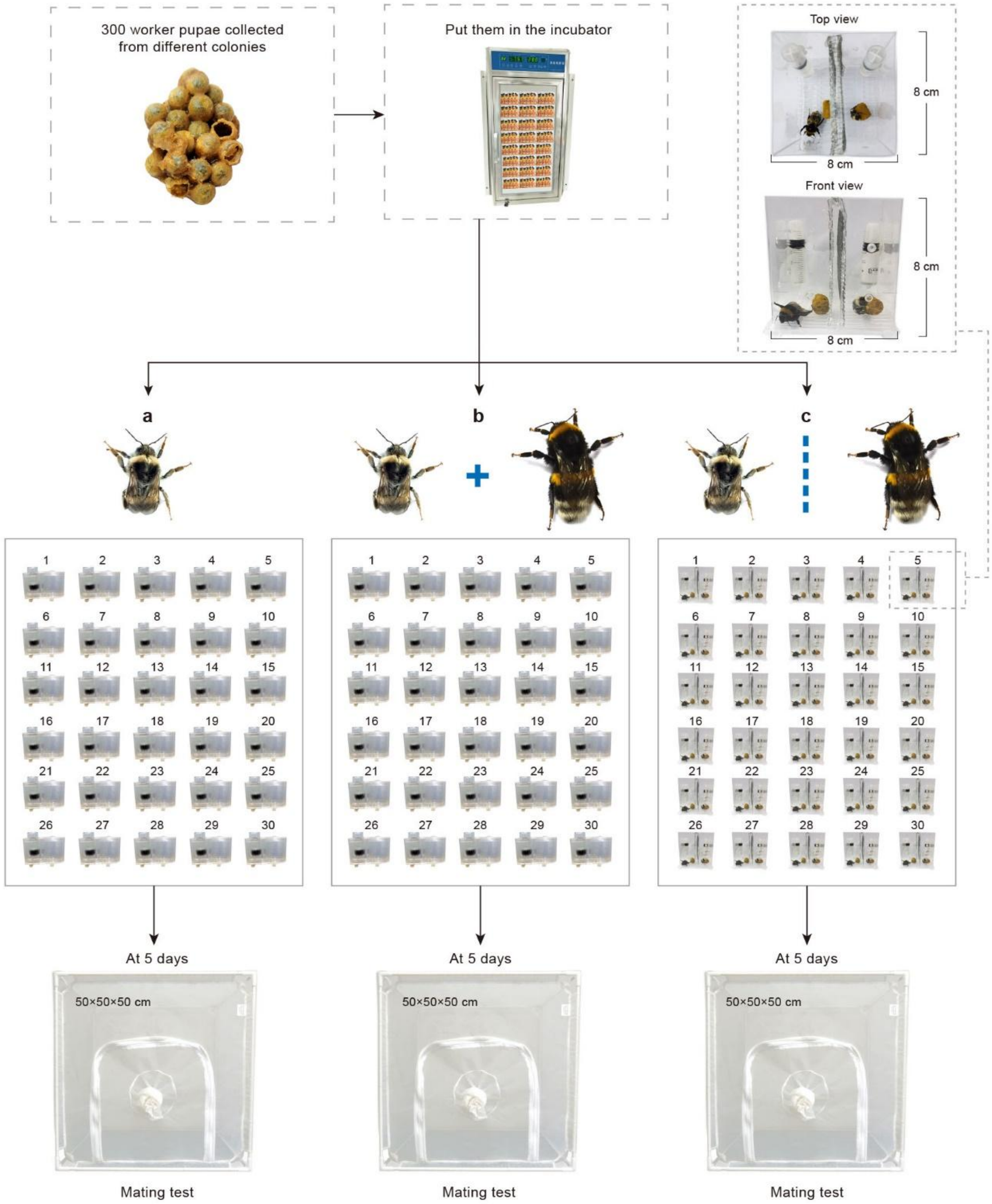

Supplementary Figure 5. The mating potential of workers is influenced by physical contact with queens. *B. terrestris* worker pupae were collected, kept individually, incubated to assist in development and monitored daily to track eclosion. Newly emerged workers were entered into one of three treatments: **a** after eclosion, each callow was kept in an individual small plastic box in solitary; **b** after eclosion, each callow was kept with one egg-laying queen, allowing physical contact between worker and queen; or **c** after eclosion, each callow was kept with one egg-laying queen but separated by a metal mesh (ϕ 1mm) that prevented physical contact. After five days, workers from each of the three treatments were exposed to mating trials.

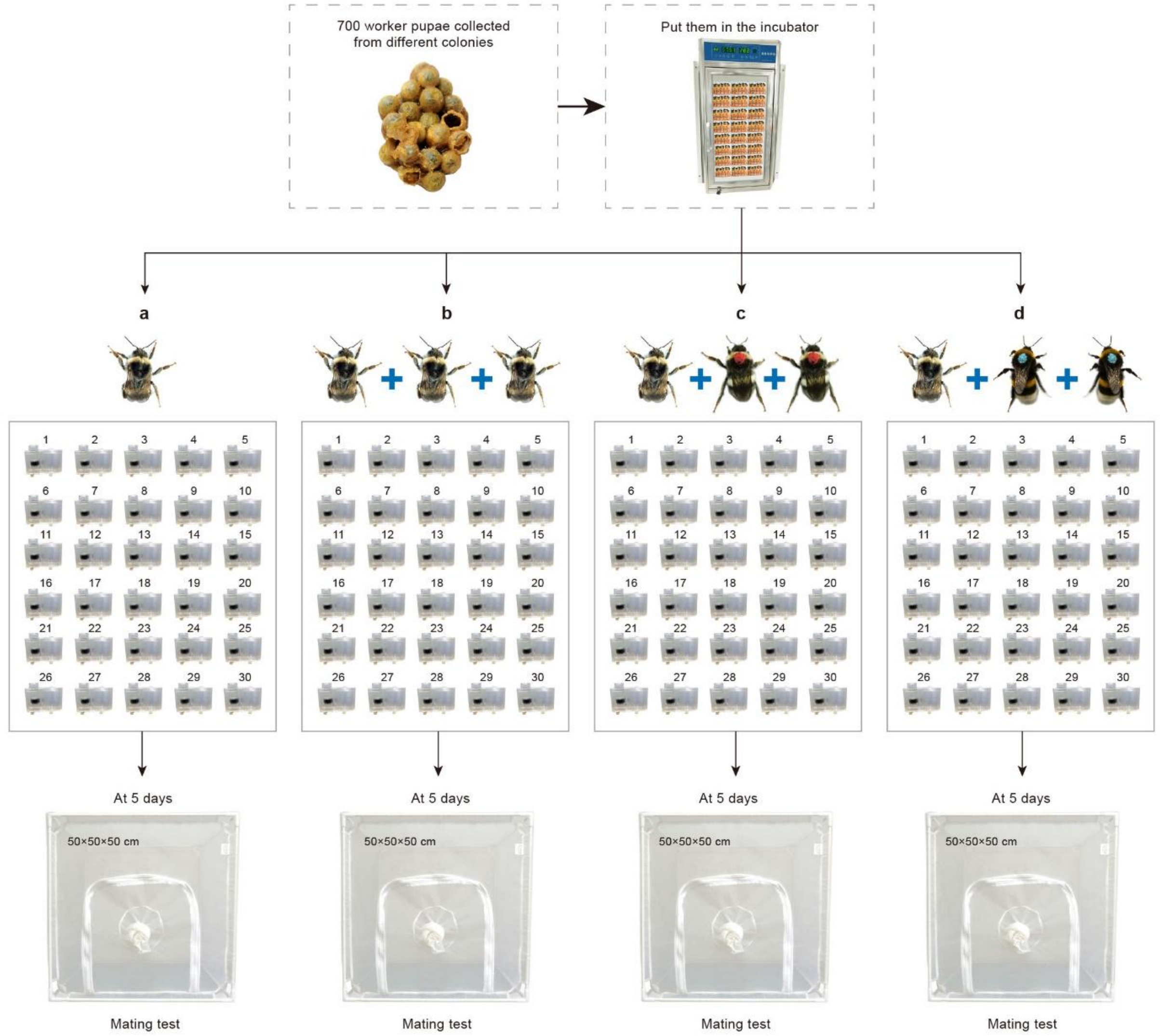

Supplementary Figure 6. Worker presence inhibits the mating ability of newly-eclosion workers. *B. terrestris* worker pupae were collected, kept individually, incubated to assist in development and monitored daily to track eclosion. Newly eclosed workers were entered into one of four treatments: **a** after eclosion, each callow worker was kept in an individual box on its own; **b** after eclosion, each of three callow workers sampled on the same day of eclosion were kept together in a single box; **c** one callow worker was kept alone in a single box for 24 hours, after which two tagged callow workers were added, and these were subsequently replaced with two new callow workers every 24 hours; and **d** after eclosion, each callow worker was kept with two tagged, egg-laying workers. After five days in their respective treatment groups, workers were exposed to mating trials.

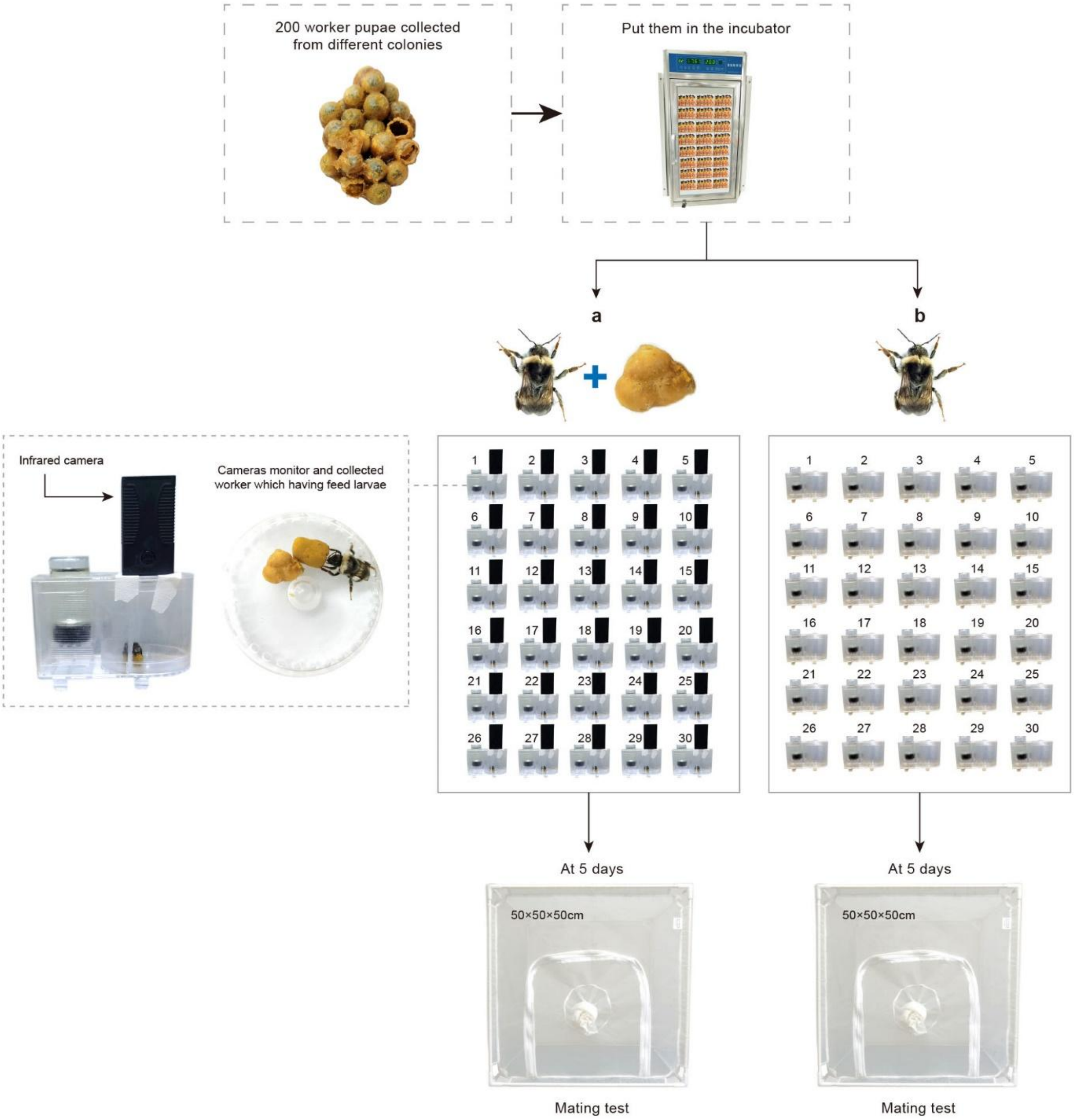

Supplementary Figure 7. Feeding behavior affects the mating ability of workers. *B. terrestris* worker pupae were collected, kept individually, incubated to assist in development and monitored daily to track eclosion. Newly eclosed callow workers were randomly assigned to one of two treatments: **a** after eclosion, each callow worker was kept in an individual box with three larvae; or **b** after eclosion, each callow was kept in an individual box alone. After five days in their respective treatment groups, workers were exposed to mating trials.

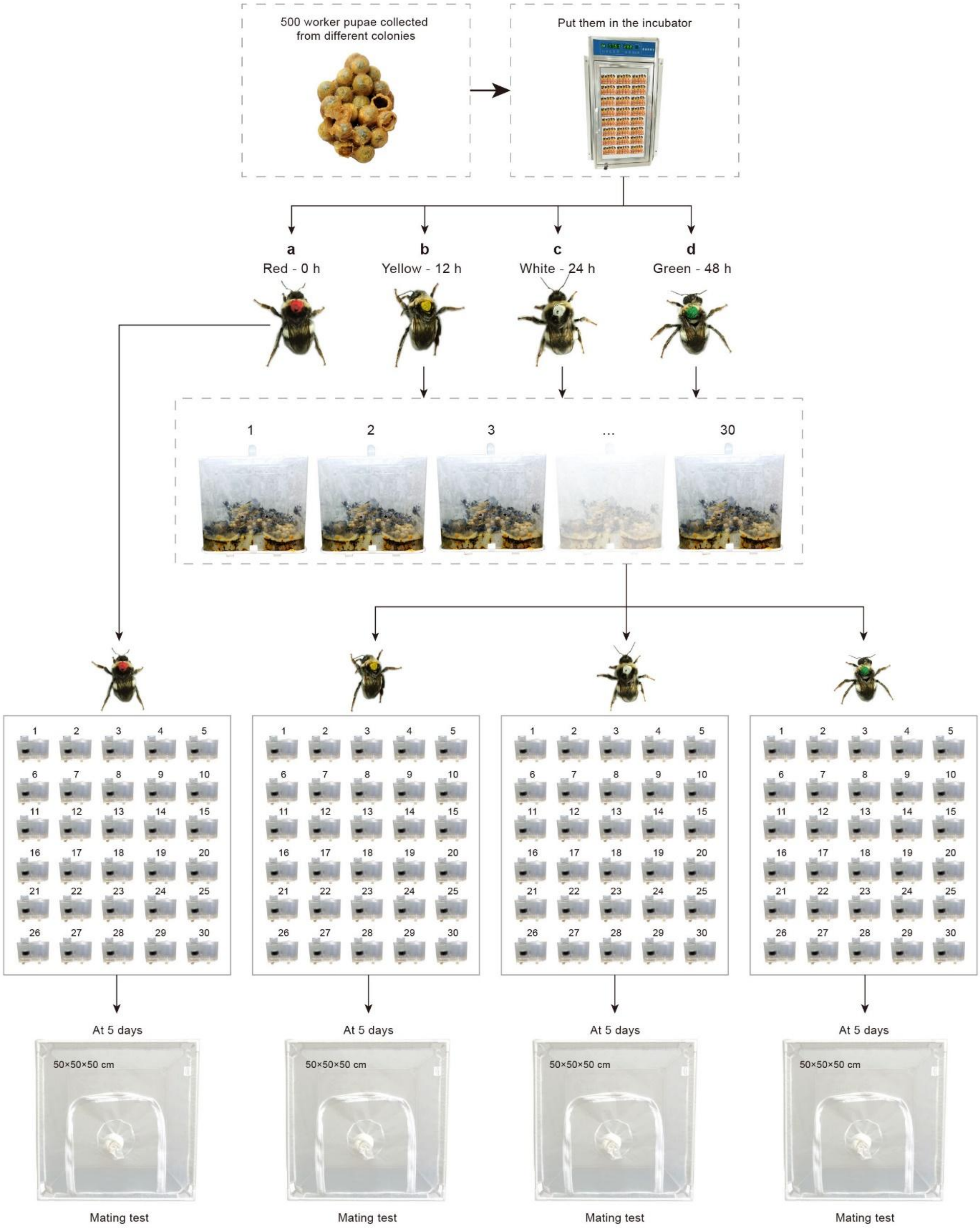

Supplementary Figure 8. The duration in a social environment post-eclosion influences mating success. We collected 360 newly emerged *B. terrestris* callow workers from previously collected pupae (sampled from 100 different colonies) and allocated them into four groups (n = 90 callow workers per group), with individuals in each group tagged with a specific color indicating the time spent in a queen-right colony post-eclosion: **a** red = 0h in social environment (i.e., no time spent in colony post-tagging); **b** yellow = 12h spent in colony post-tagging; **c** white = 24h spent in colony post-tagging; and **d** green = 48h spent in colony post-tagging. For each treatment group, 30 workers were randomly subsampled, and after social exposure each bee was kept in an individual box. When they were 5 days old, workers were exposed to mating trials.

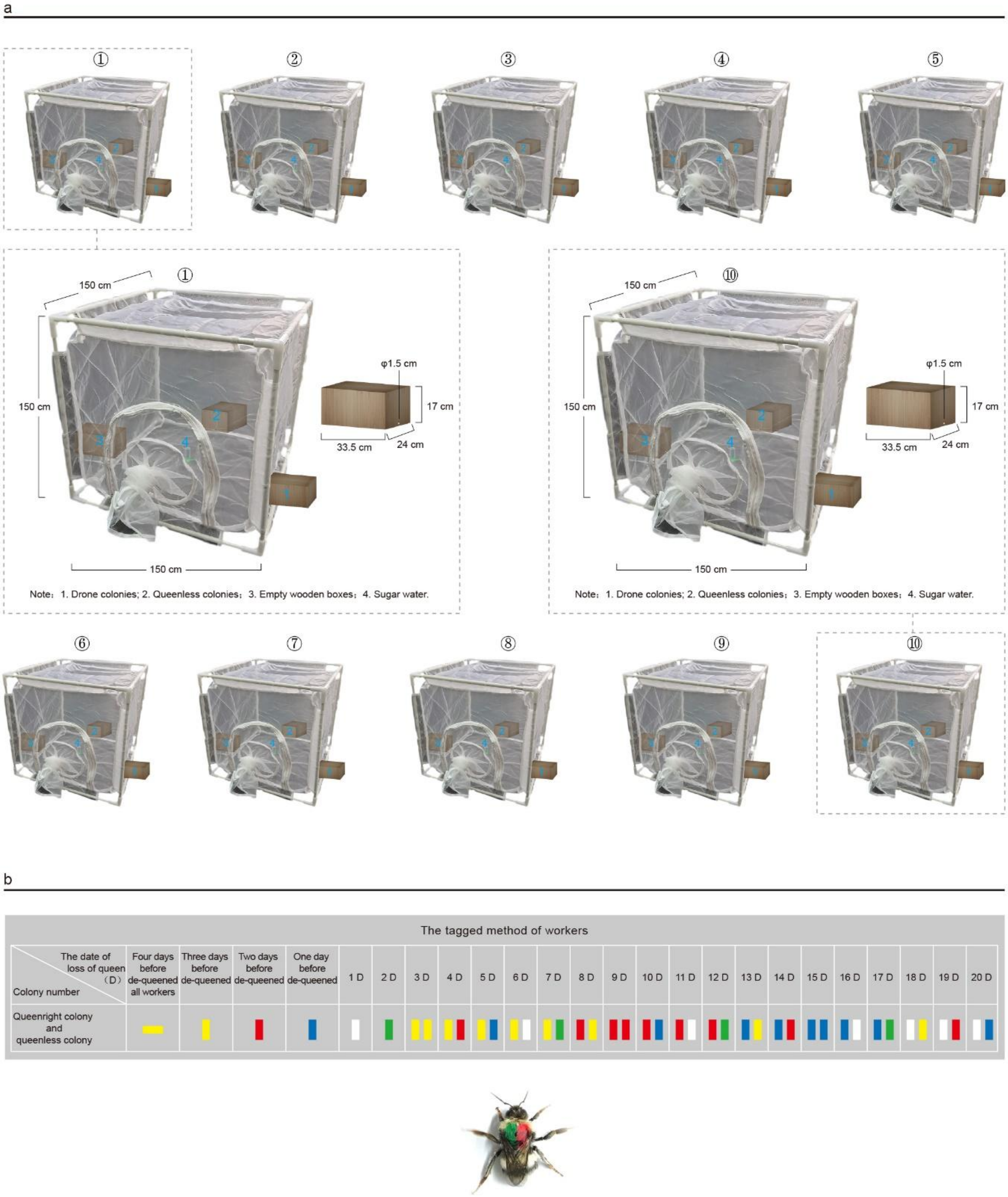

Supplementary Figure 9. Queen-loss enables worker mating under semi-field conditions. **a** For this experiment, we used ten *B. terrestris* queen-right colonies that were randomly allocated into one of two treatments: dequeened colonies, here outlined as test cages numbered: ① ② ③ ④ ⑤; and queenright colonies with test cages numbered: ⑥ ⑦ ⑧ ⑨ ⑩. For the purposes of the mating exposure, each colony was placed in a mating cage, which was supplied with three boxes, a worker-producing colony, a male-producing colony, and an empty colony box. **b** Prior to manipulation, for each colony, we collected workers and callow workers and marked them with colors and patterns to distinguish their day of eclosion. We performed this for a further three days before we started the experiment. We next removed queens from five colonies, and for all ten colonies, collected and tagged newly eclosed callow workers for twenty days. Callow workers received a unique color pattern to indicate their respective day of eclosion.

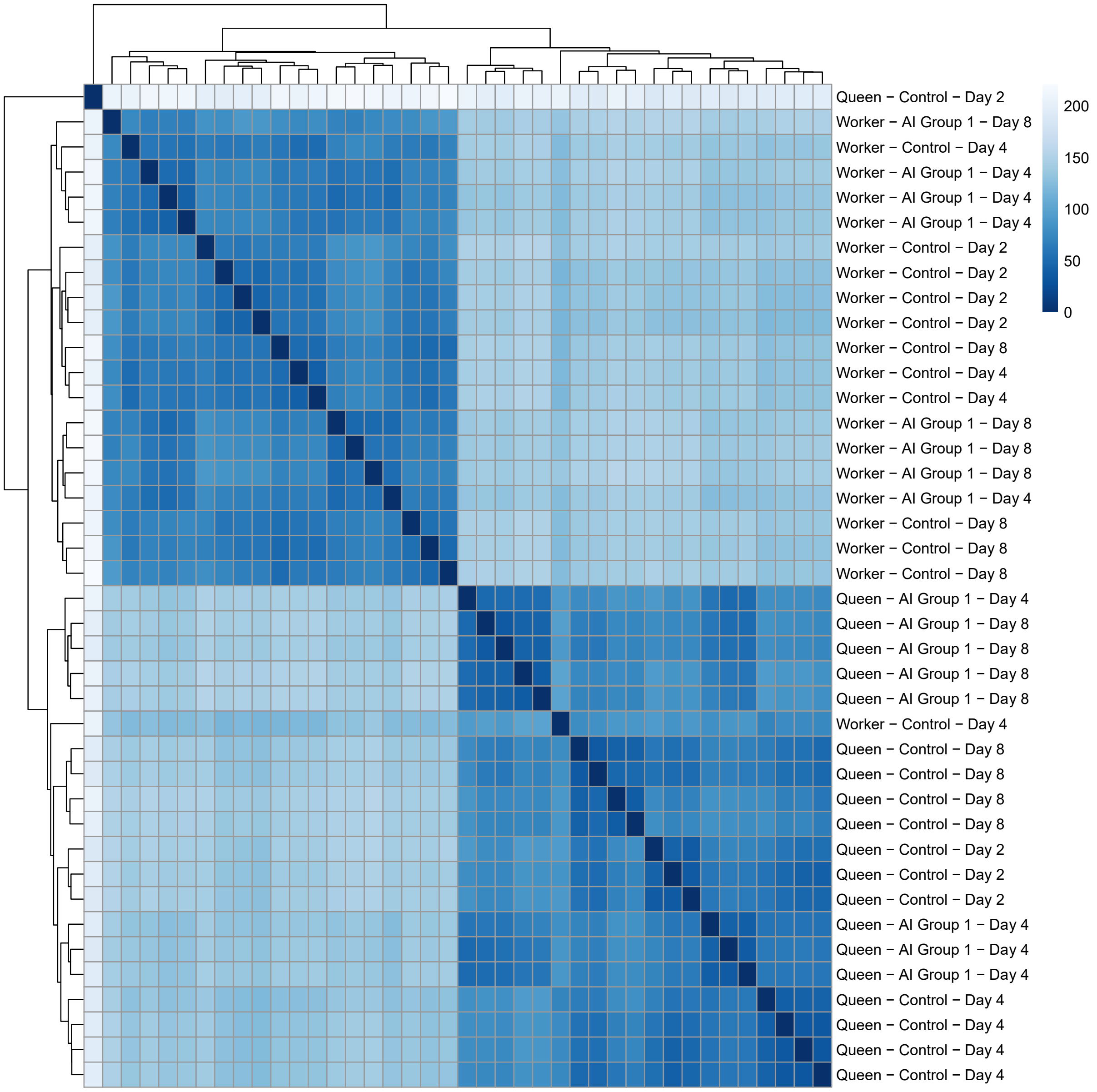

Supplementary Figure 10. Hierarchical clustering of reproductive tissue (spermatheca, vagina, and median oviduct) samples extracted from inseminated and control bumble bee castes based on gene expression profiles. For each *B. terrestris* sample, the caste, the status of insemination (AI Group 1 = Artificial insemination; Control = non-inseminated) and age at collection post-treatment are provided.

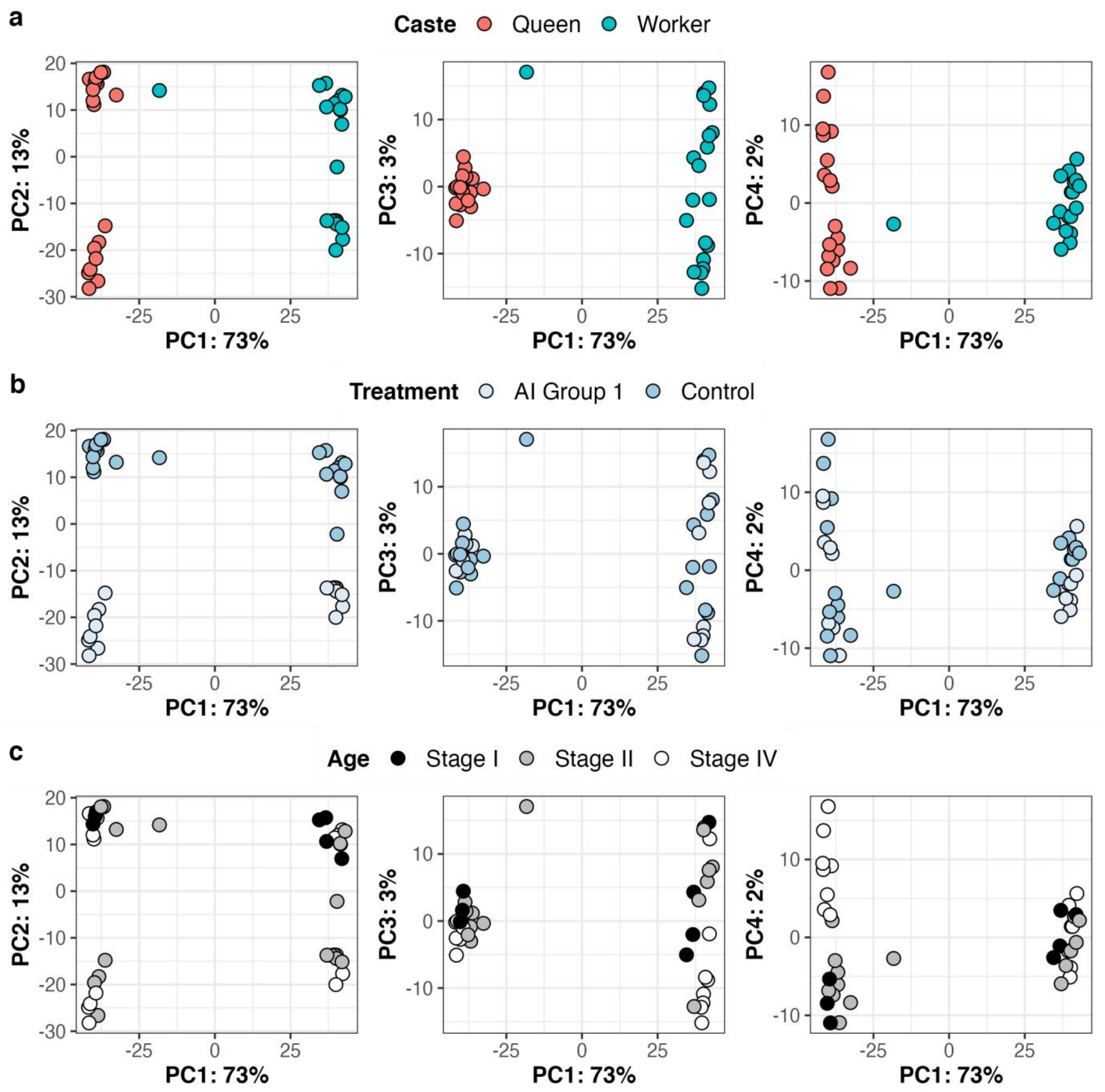

Supplementary Figure 11. Principal component analysis of gene expression profiles for the reproductive tissues (spermatheca, vagina, and median oviduct) of workers and queens. Scatterplots displaying different combinations for the first principal components with that of the second, third and fourth, with individual plots colored by: **a** caste (*B. terrestris* queen or worker); **b** treatment (AI Group 1 = Artificial insemination; Control = non-inseminated); and **c** age (Day 2 = two days post-treatment; Day 4 = four days post-treatment; Day 8 = eight days post-treatment).

a

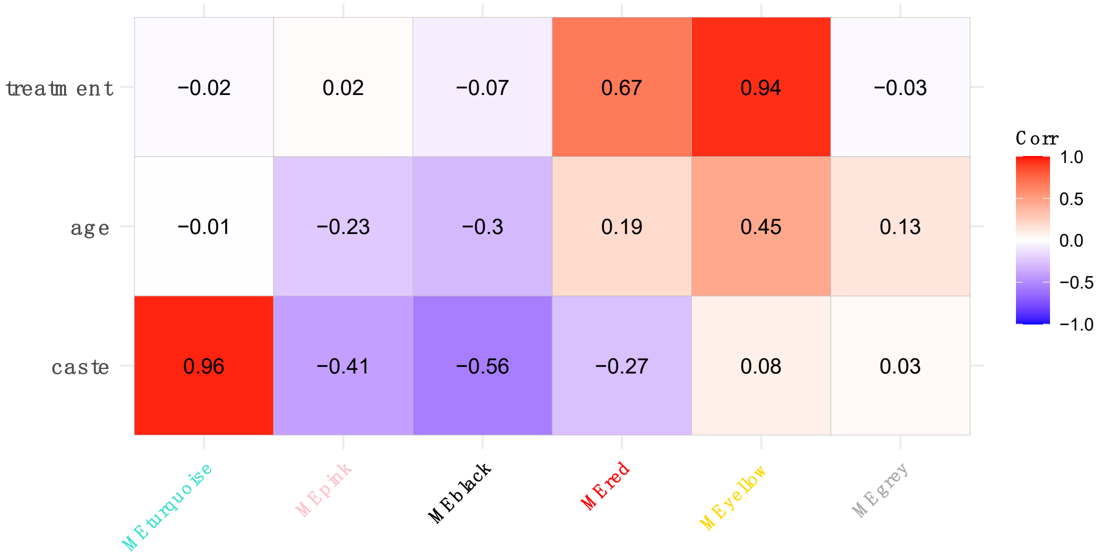

b

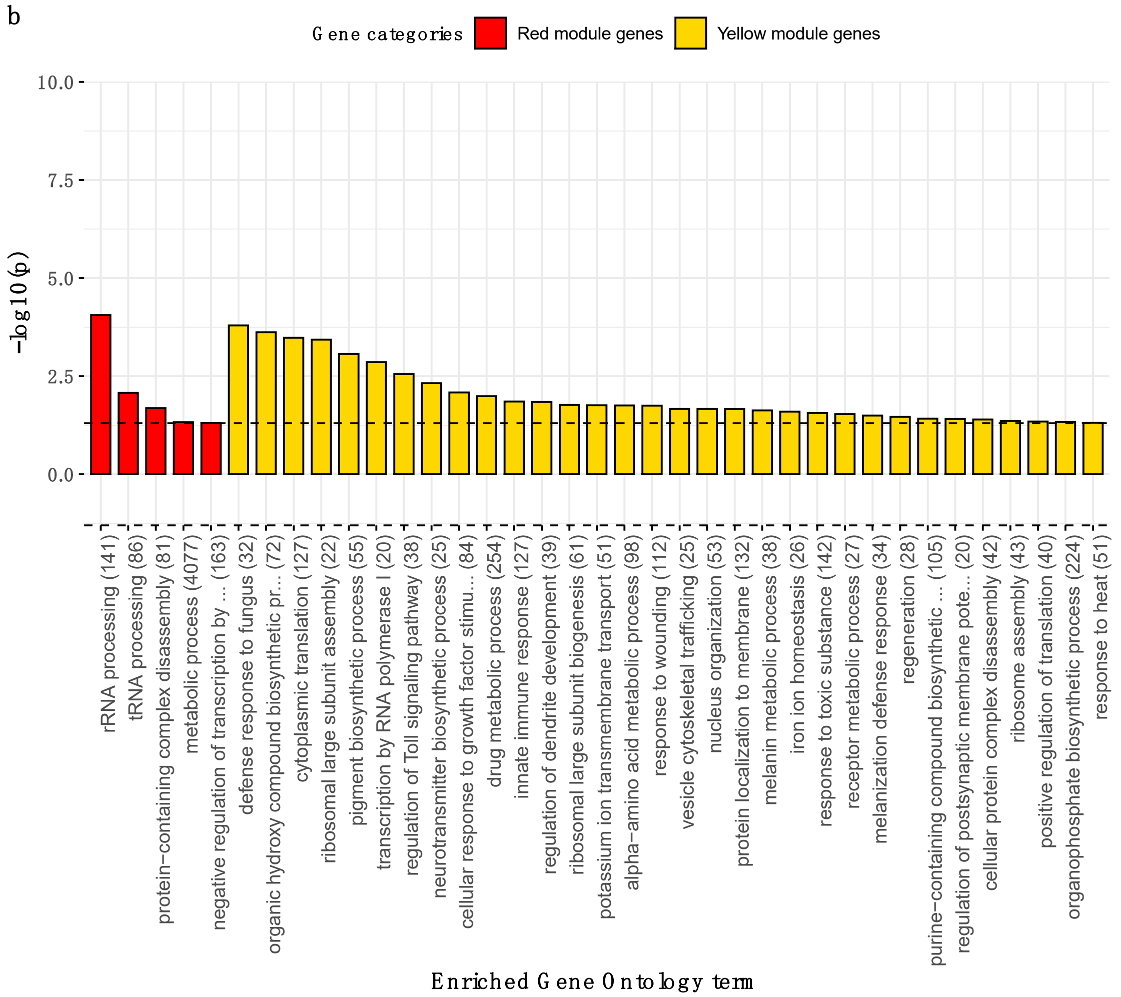

Supplementary Figure 12. Weighted co-expression network analysis for reproductive tissues of bumble bee (*B. terrestris*) castes. **a** Heatmap displaying correlations between modules (gene clusters) identified by WGCNA and each of caste, stage (age post-insemination treatment) and insemination treatment. **b** Bar chart for gene ontology terms significantly enriched (one-sided Fisher's exact test; $p < 0.05$; node size = 20) for genes with elevated members for modules "red" and "yellow", which

strongly correlated with treatment. Each GO term category (BP: Biological Process; MF: Molecular Function; CC: Cellular Component) is designated by an individual color. On the y-axis, we provide each enriched GO term, the GO term description, as well as the number of genes in the *B. terrestris* genome assembly annotated with each term. We provide a measure of significance (-log10 transformed *p* values) on the x-axis with a dashed, black line indicating the threshold of significance (-log10(p = 0.05)).

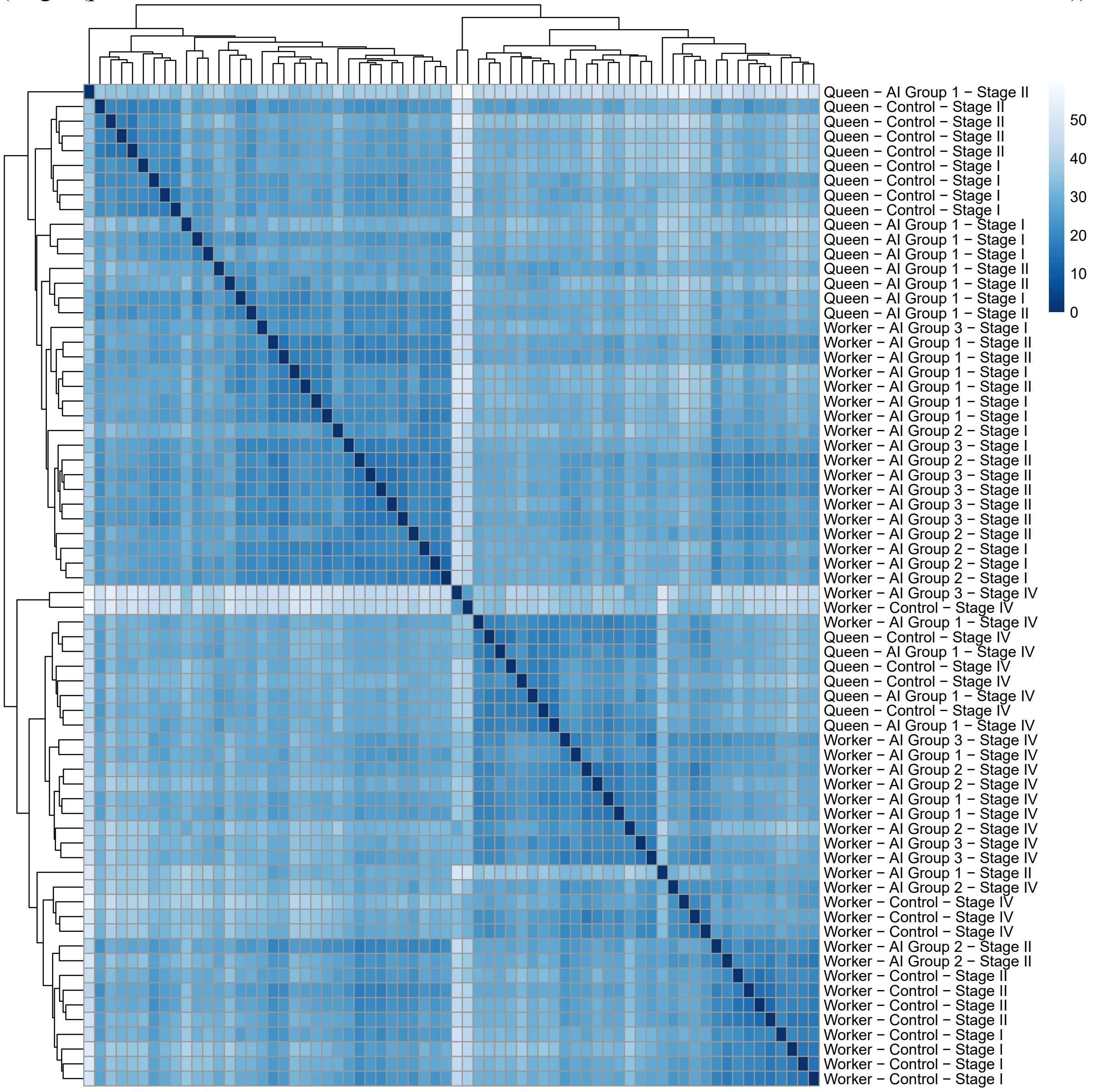

Supplementary Figure 13. Hierarchical clustering of brain samples extracted from inseminated and control bumble bee castes based on gene expression profiles. For each *B. terrestris* sample, the caste, the status of insemination (AI Group 1 = Artificial insemination, including semen; AI Group 2 = Artificial insemination, with only diluent, no semen; AI Group 3 = Injection, without insemination; Control = non-inseminated) and the stage of ovarian development (Stage I = immature ovary with a thread-like appearance; Stage II = nutritive cell (i.e., nurse cell) larger than egg cell; Stage IV = mature egg (after oviposition) is provided.

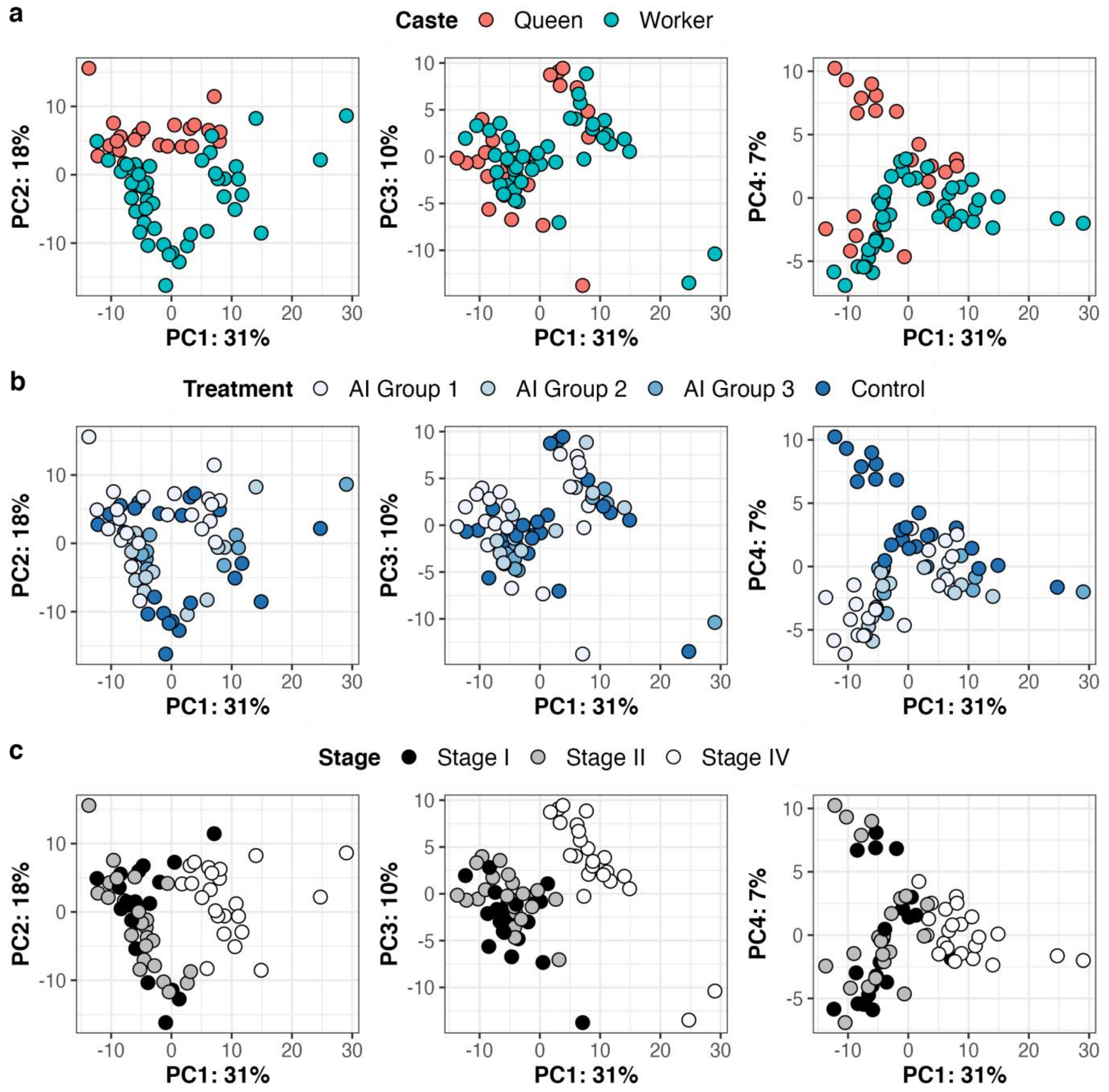

Supplementary Figure 14. Principal component analysis of gene expression profiles for the brains of *B. terrestris* workers and queens. Scatterplots displaying different combinations for the first principal components with that of the second, third and fourth, with individual plots colored by: **a** caste (queen or worker); **b** treatment (AI Group 1 = Artificial insemination, including semen; AI Group 2 = Artificial insemination, with only diluent, no semen; AI Group 3 = Injection, without insemination; Control = non-inseminated); and **c** stage of ovarian development (Stage I = immature ovary with a thread-like appearance; Stage II = nutritive cell (i.e., nurse cell) larger than egg cell; Stage IV = mature egg (after oviposition).

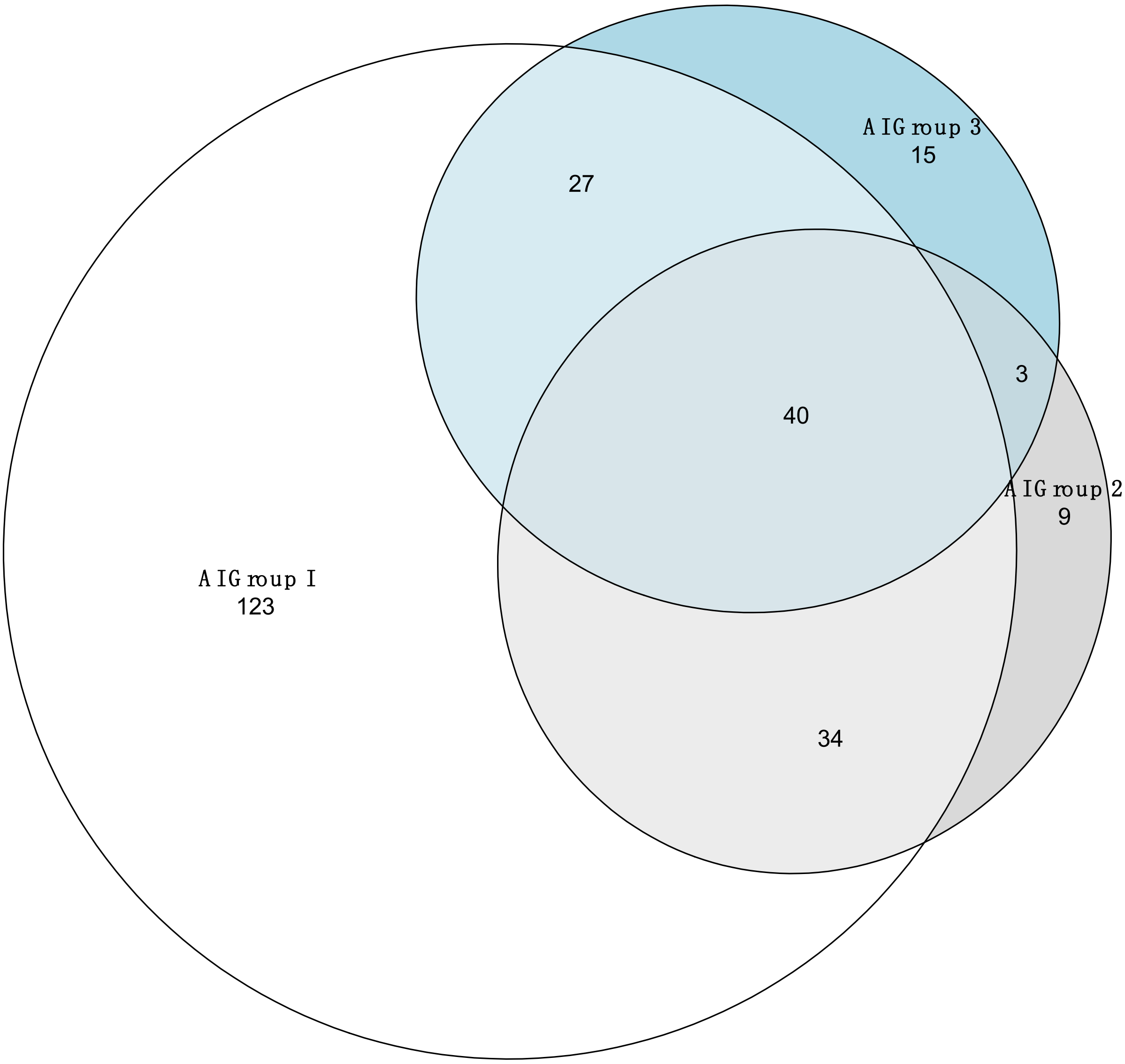

Supplementary Figure 15. Genes uniquely differentially expressed in the brains of inseminated *B. terrestris* workers in comparison to *B. terrestris* control workers. Euler plot displaying the conserved but also treatment-specific significantly differentially expressed genes (DEGs) (Likelihood ratio test: FDR < 0.05) between each of three treatment groups (AI Group 1 = Artificial insemination, including semen; AI Group 2 = Artificial insemination, with only diluent, no semen; AI Group 3 = Injection, without insemination) and control (non-inseminated) workers.

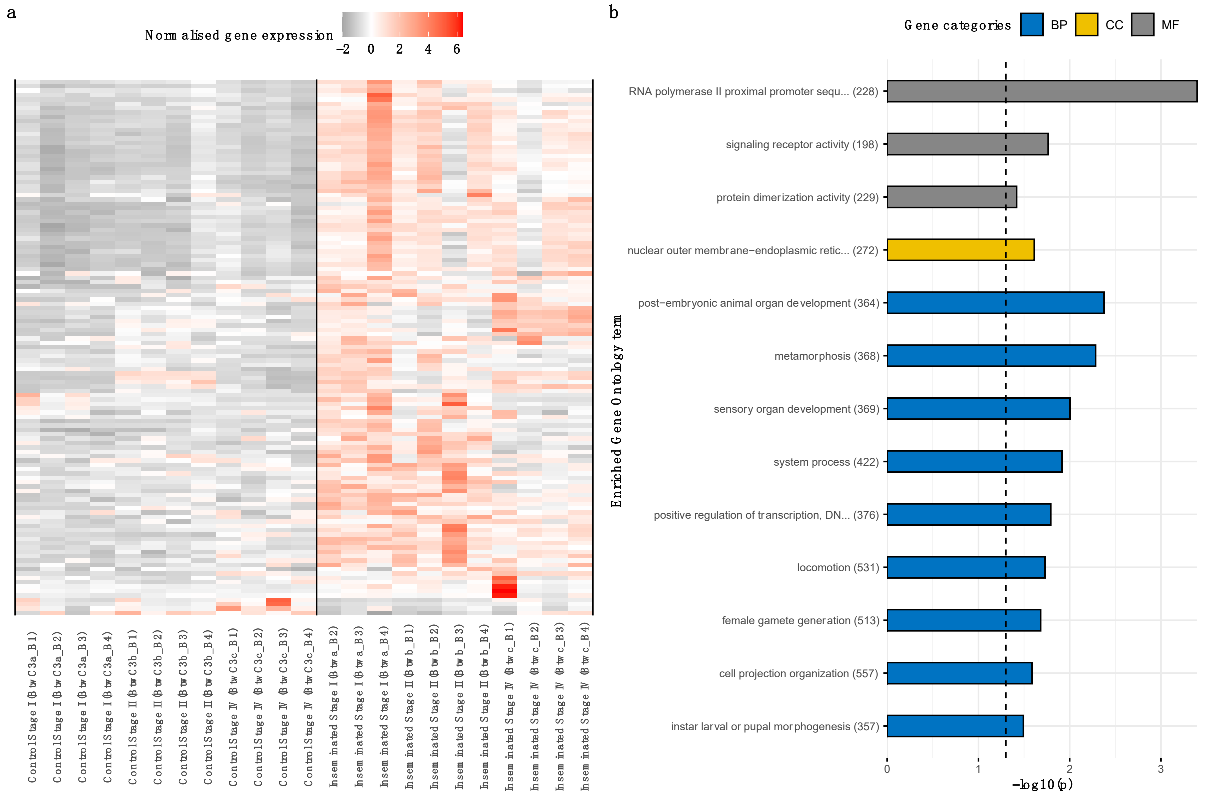

Supplementary Figure 16. Differentially expressed genes within the brains of inseminated and non-inseminated *B. terrestris* workers. a Heatmap displaying expression profiles of significantly differentially genes (Likelihood ratio test; FDR < 0.05) uniquely identified between the brains of artificially inseminated and control workers. For each gene, we provide normalized (scaled) gene-level expression to highlight relative differences in expression between treatment groups. **b** Bar chart for gene ontology (GO) terms significantly enriched (one-sided Fisher's exact test; $P < 0.05$; node size = 50) for differentially expressed genes uniquely elevated in inseminated bees compared to control. Each GO term category (BP: Biological Process; MF: Molecular Function; CC: Cellular Component) is designed by an individual color. On the y-axis, we provide each enriched GO term, the GO term description, as well as the number of genes in the *B. terrestris* genome assembly annotated with each term. We provide a measure of significance (-log10 transformed *P* values) on the x-axis with a dashed, black line indicating threshold of significance (-log10(P = 0.05)). Full results of the GO term enrichment analyses are provided in Supplementary Data 8.

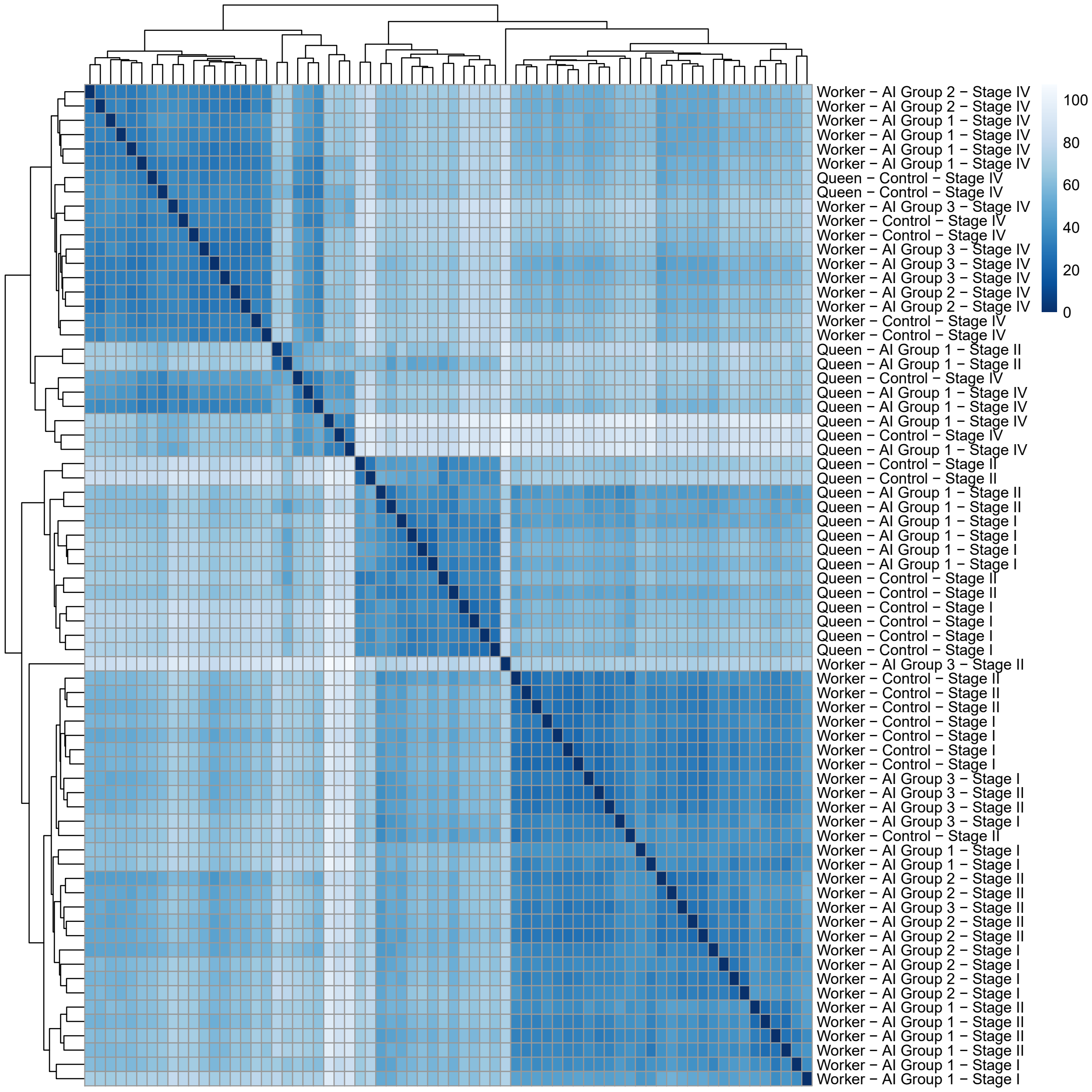

Supplementary Figure 17. Hierarchical clustering of fat body samples extracted from inseminated and control bumble bee castes based on gene expression profiles. For each *B. terrestris* sample, the caste, the status of insemination (AI Group 1 = Artificial insemination, including semen; AI Group 2 = Artificial insemination, with only diluent, no semen; AI Group 3 = Injection, without insemination; Control = non-inseminated) and the stage of ovarian development (Stage I = immature ovary with a thread-like appearance; Stage II = nutritive cell (i.e., nurse cell) larger than egg cell; Stage IV = mature egg (after oviposition) is provided.

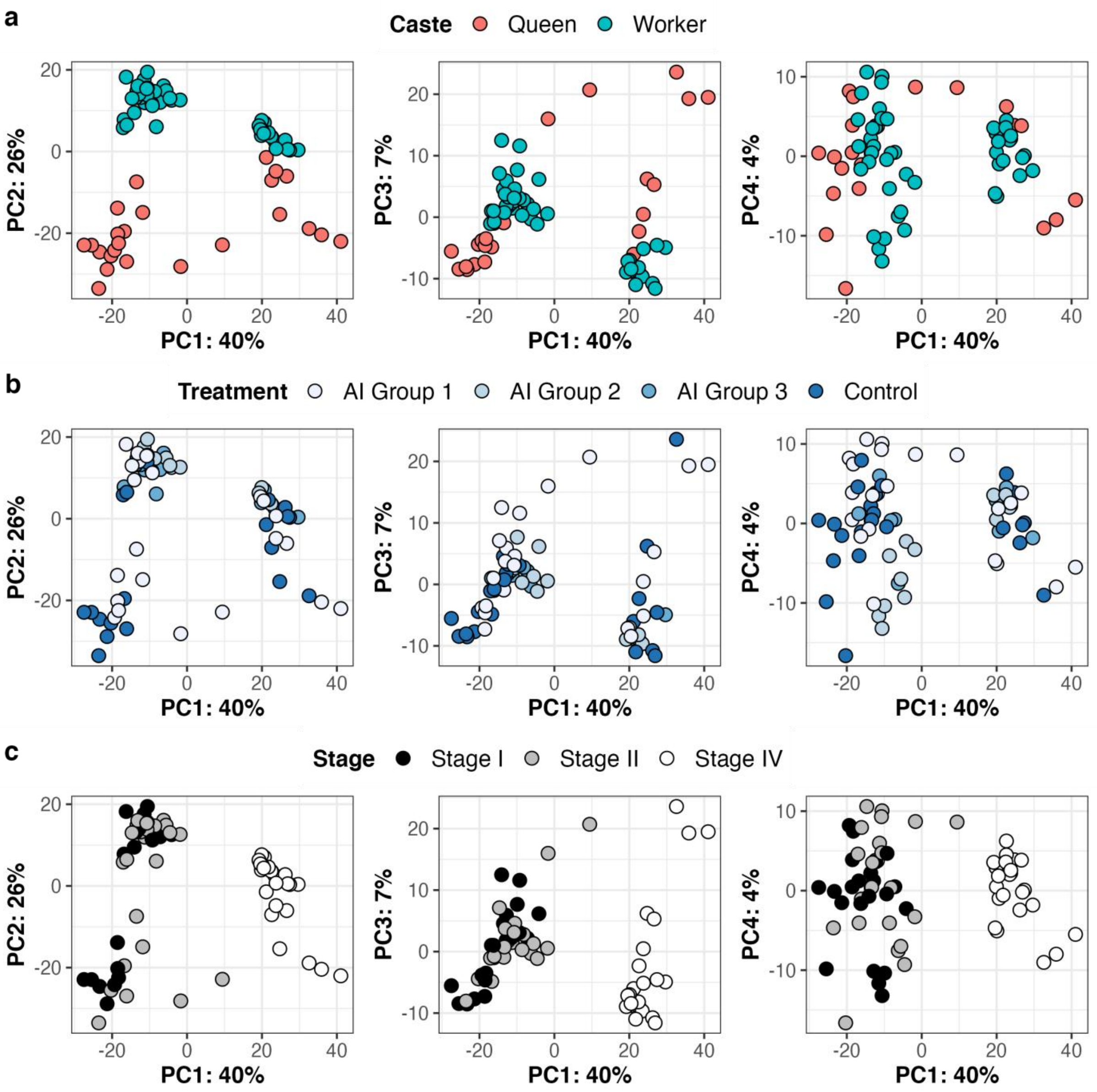

Supplementary Figure 18. Principal component analysis of gene expression profiles for the fat bodies of *B. terrestris* workers and queens. Scatterplots displaying different combinations for the first principal components with that of the second, third and fourth, with individual plots colored by: **a** caste (queen or worker); **b** treatment (AI Group 1 = Artificial insemination, including semen; AI Group 2 = Artificial insemination, with only diluent, no semen; AI Group 3 = Injection, without insemination; Control = non-inseminated); and **c** stage of ovarian development (Stage I = immature ovary with a thread-like appearance; Stage II = nutritive cell (i.e., nurse cell) larger than egg cell; Stage IV = mature egg (after oviposition)).

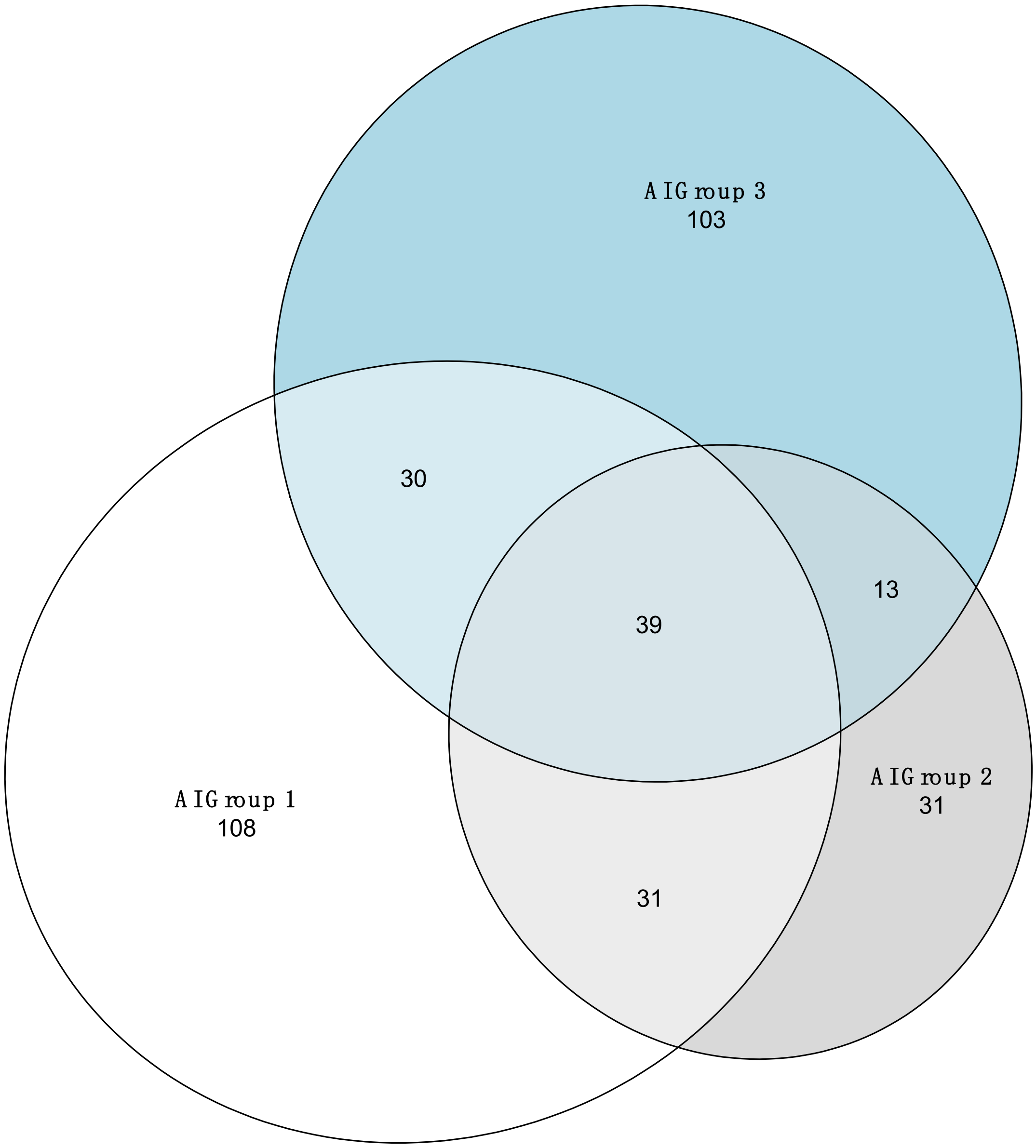

Supplementary Figure 19. Genes uniquely differentially expressed in the fat body of inseminated *B. terrestris* workers in comparison to control *B. terrestris* workers. Euler plot displaying the conserved but also treatment-specific significantly differentially expressed genes (DEGs) (Likelihood ratio test: FDR < 0.05) between each of three treatment groups (AI Group 1 = Artificial insemination, including semen; AI Group 2 = Artificial insemination, with only diluent, no semen; AI Group 3 = Injection, without insemination) and control (non-inseminated) workers.

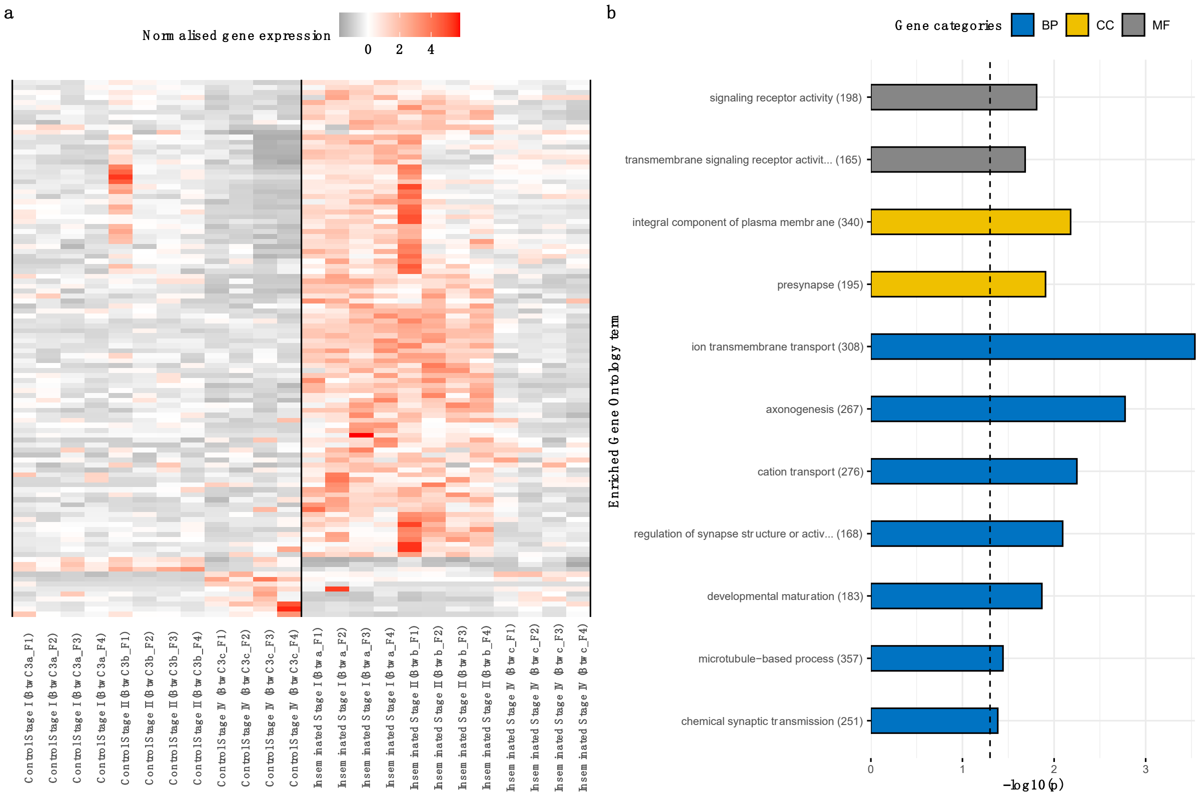

Supplementary Figure 20. Differentially expressed genes within the fat body of inseminated and non-inseminated *B. terrestris* workers. a Heatmap displaying expression profiles of significantly differentially genes (Likelihood ratio test; FDR < 0.05) uniquely identified between the fat body of artificially inseminated and control workers. For each gene, we provide normalized (scaled) gene-level expression to highlight relative differences in expression between treatment groups. **b** Bar chart for gene ontology (GO) terms significantly enriched (one-sided Fisher's exact test; $P < 0.05$; node size = 50) for differentially expressed genes uniquely elevated in inseminated bees compared to control. Each GO term category (BP: Biological Process; MF: Molecular Function; CC: Cellular Component) is designed by an individual color. On the y-axis, we provide each enriched GO term, the GO term description, as well as the number of genes in the *B. terrestris* genome assembly annotated with each term. We provide a measure of significance (-log10 transformed *p* values) on the x-axis with a dashed, black line indicating threshold of significance (-log10(P = 0.05)). Full results of the GO term enrichment analyses are provided in Supplementary Data 8.

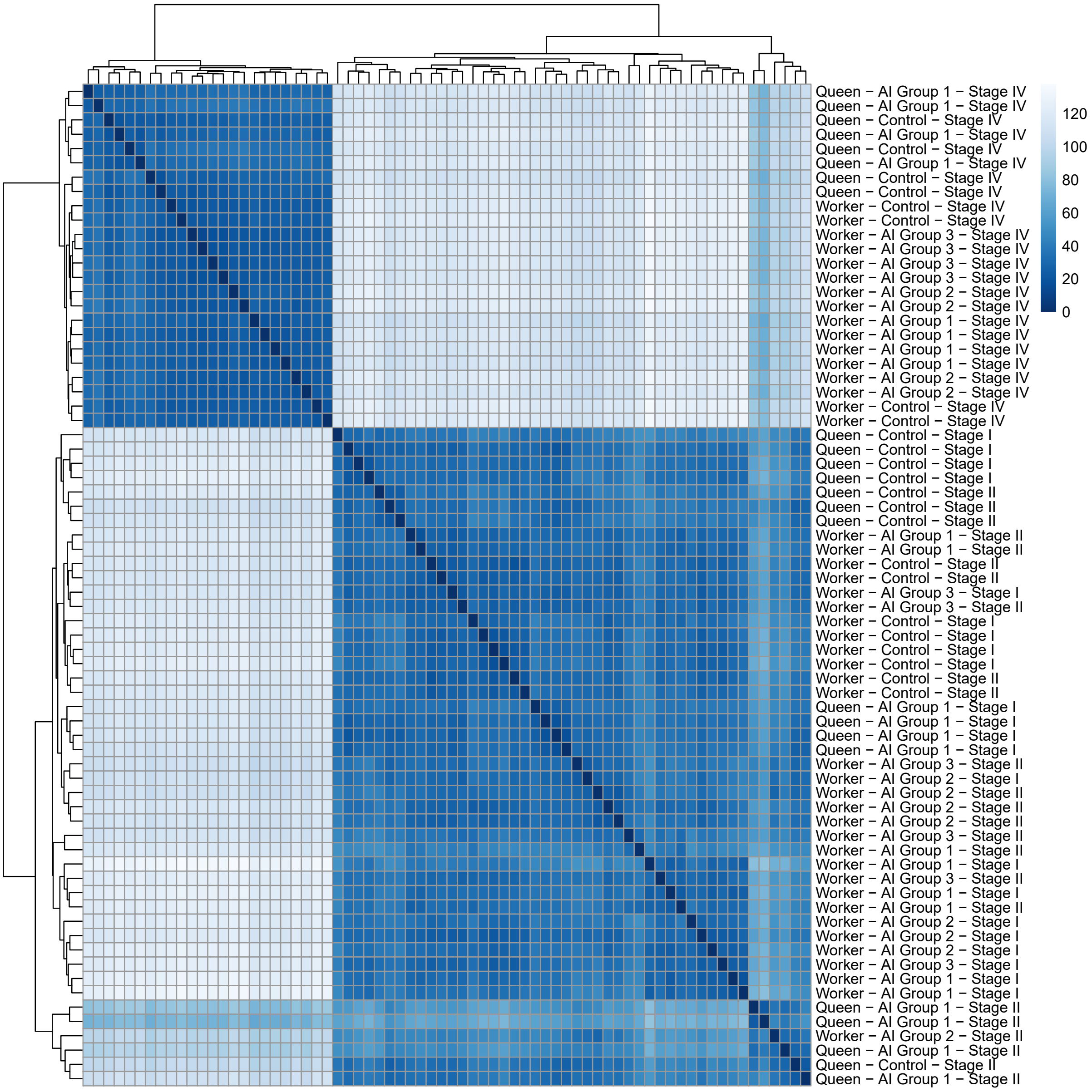

Supplementary Figure 21. Hierarchical clustering of ovarian samples extracted from inseminated and control bumble bee (*B. terrestris*) castes based on gene expression profiles. For each sample, the caste, the status of insemination (AI Group 1 = Artificial insemination, including semen; AI Group 2 = Artificial insemination, with only diluent, no semen; AI Group 3 = Injection, without insemination; Control = non-inseminated) and the stage of ovarian development (Stage I = immature ovary with a thread-like appearance; Stage II = nutritive cell (i.e., nurse cell) larger than egg cell; Stage IV = mature egg (after oviposition) is provided.

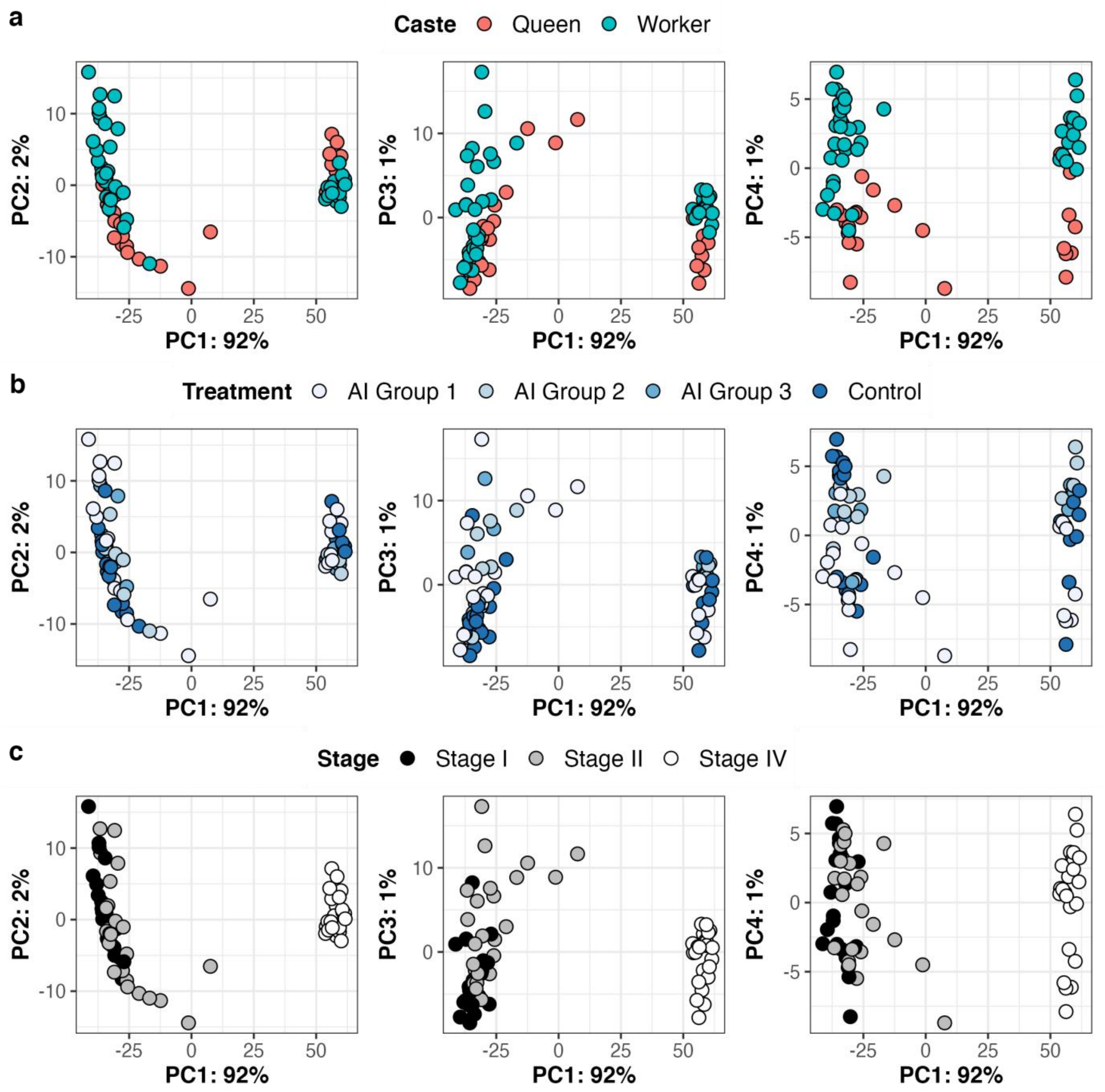

Supplementary Figure 22. Principal component analysis of gene expression profiles for the ovaries of *B. terrestris* workers and queens. Scatterplots displaying different combinations for the first principal components with that of the second, third and fourth, with individual plots colored by: **a** caste (queen or worker); **b** treatment (AI Group 1 = Artificial insemination, including semen; AI Group 2 = Artificial insemination, with only diluent, no semen; AI Group 3 = Injection, without insemination; Control = non-inseminated); and **c** stage of ovarian development (Stage I = immature ovary with a thread-like appearance; Stage II = nutritive cell (i.e., nurse cell) larger than egg cell; Stage IV = mature egg (after oviposition)).

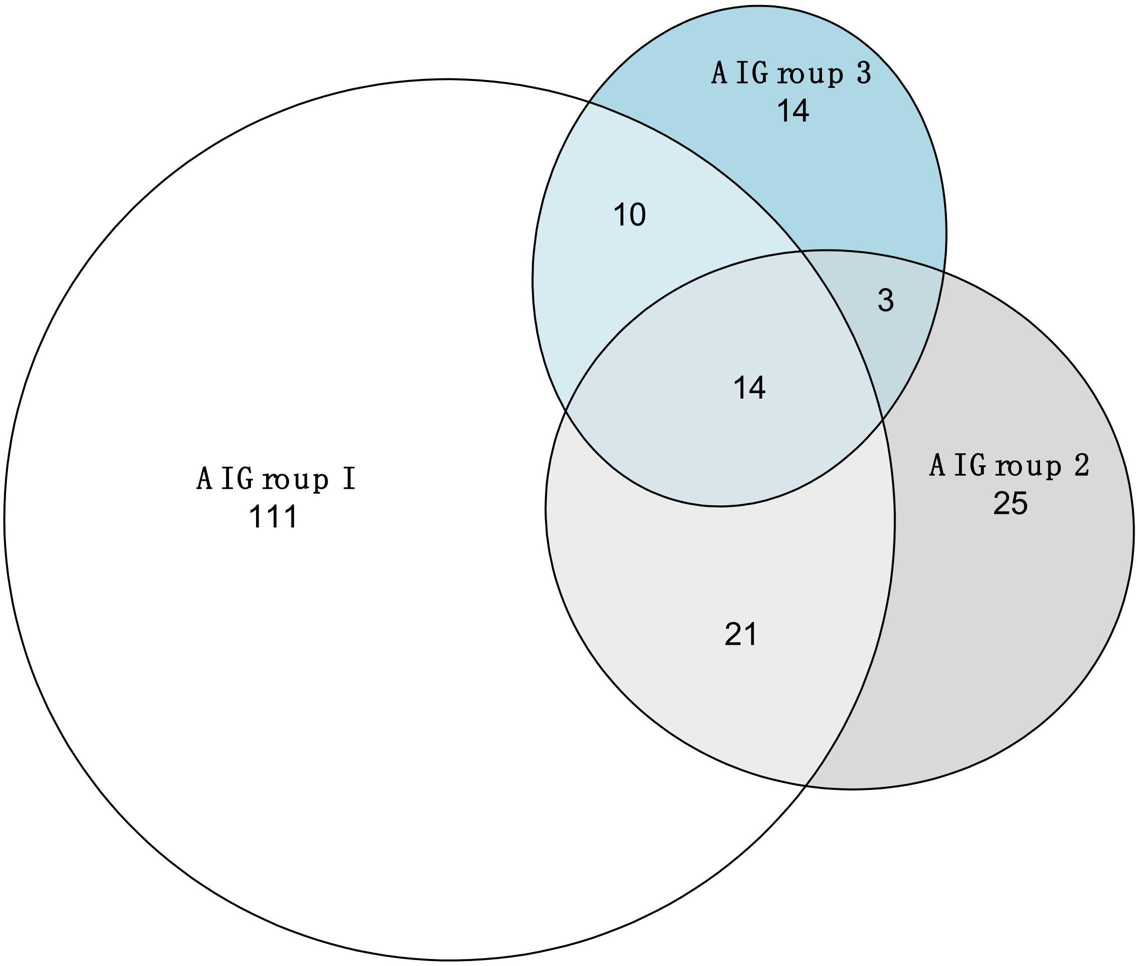

Supplementary Figure 23. Genes uniquely differentially expressed in the ovaries of inseminated *B. terrestris* workers in comparison to control *B. terrestris* workers. Euler plot displaying the conserved but also treatment-specific significantly differentially expressed genes (DEGs) (Likelihood ratio test: FDR < 0.05) between each of three treatment groups (AI group 1 = Artificial insemination, including semen; AI group 2 = Artificial insemination, with only diluent, no semen; AI group 3 = Injection, without insemination) and control (non-inseminated) workers.

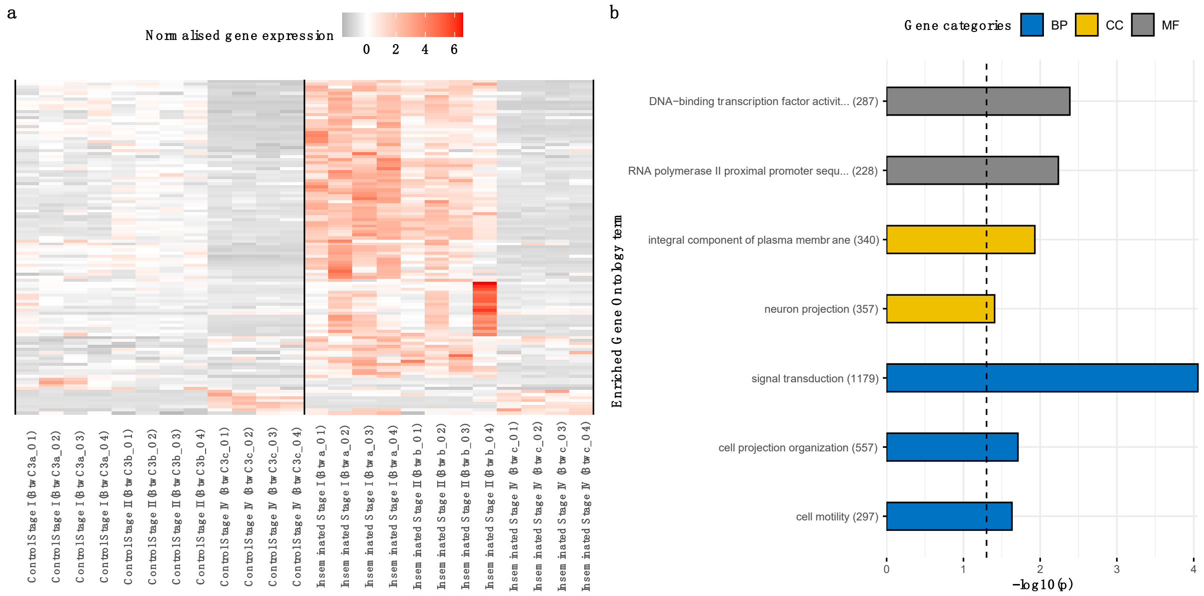

Supplementary Figure 24. Differentially expressed genes within the ovaries of inseminated and non-inseminated *B. terrestris* workers. a Heatmap displaying expression profiles of significantly differentially genes (Likelihood ratio test; FDR < 0.05) uniquely identified between the ovaries of artificially inseminated and control workers. For each gene, we provide normalized (scaled) gene-level expression to highlight relative differences in expression between treatment groups. **b** Bar chart for gene ontology (GO) terms significantly enriched (one-sided Fisher's exact test; $P < 0.05$; node size = 50) for differentially expressed genes uniquely elevated in inseminated bees compared to control. Each GO term category (BP: Biological Process; MF: Molecular Function; CC: Cellular Component) is designed by an individual color. On the y-axis, we provide each enriched GO term, the GO term description, as well as the number of genes in the *B. terrestris* genome assembly annotated with each term. We provide a measure of significance (-log10 transformed P values) on the x-axis with a dashed, black line indicating threshold of significance (-log10(P = 0.05)). Full results of the GO term enrichment analyses are provided in Supplementary Data 8.

References

1. Chen, S., Zhou, Y., Chen, Y. & Gu, J. Fast: An ultra-fast all-in-one FASTQ preprocessor. *Bioinformatics* **34**, i884–i890 (2018).
2. Dobin, A. et al. STAR: ultrafast universal RNA-seq aligner. *Bioinformatics* **29**, 15–21 (2013).
3. Love, M.I., Huber, W. & Anders, S. Moderated estimation of fold change and dispersion for RNA-seq data with DESeq2. *Genome Biol.* **15**, 550 (2014).
