# Supplementary material for: Unexpected worker mating and colony-founding in a superorganism
Source: Nat Commun. 2023 Sep 7;14:5499. doi: 10.1038/s41467-023-41198-6 (PMC10484907; doi:10.1038/s41467-023-41198-6)
Supplement: Supplementary file 3 — Description of Additional Supplementary Files [file 41467_2023_41198_MOESM3_ESM.pdf]

Description of Additional Supplementary Files

Supplementary Data 1: Primers for microsatellite-based polymorphism analysis.

Supplementary Data 2: Polymorphism analysis for each of eight microsatellite loci in bumble bees.

Supplementary Data 3: The sampling number of bumble bee workers from AI colonies.

Supplementary Data 4: The average exclusion probability of first parent（E-1P), second parent (E-2P), and parent pair (E-PP) based on eight microsatellite loci.

Supplementary Data 5: Microsatellite loci and paternity test results of five AI worker-produced colonies A, B, C, D and E.

Supplementary Data 6: Microsatellite genotypes of five AI worker-produced colonies A, B, C, D and E.

Supplementary Data 7: Sampling groups for tissues for transcriptomic-based analysis.

Supplementary Data 8: Output files for gene count estimation, differential gene expression analysis, and GO term enrichment analysis of inseminated and control bumble bee castes.

Supplementary Movie 1: Worker callow feeding larvae in colony. Within 24 hours after emergence, and prior to wing maturation, callow workers began to assist the queen in feeding the larvae.
