# Supplementary material for: Unexpected worker mating and colony-founding in a superorganism
Source: Nat Commun. 2023 Sep 7;14:5499. doi: 10.1038/s41467-023-41198-6 (PMC10484907; doi:10.1038/s41467-023-41198-6)
Supplement: Supplementary file 8 — Reporting Summary [file 41467_2023_41198_MOESM8_ESM.pdf]

nature portfolio

Corresponding author(s): Jilian Li and Mark JF Brown

Last updated by author(s): Feb 13, 2023

Reporting Summary

Nature Portfolio wishes to improve the reproducibility of the work that we publish. This form provides structure for consistency and transparency in reporting. For further information on Nature Portfolio policies, see our Editorial Policies and the Editorial Policy Checklist.

Statistics

For all statistical analyses, confirm that the following items are present in the figure legend, table legend, main text, or Methods section.

n/a	Confirmed	
☐	☒	The exact sample size (*n*) for each experimental group/condition, given as a discrete number and unit of measurement
☐	☒	A statement on whether measurements were taken from distinct samples or whether the same sample was measured repeatedly
☐	☒	The statistical test(s) used AND whether they are one- or two-sided<br>*Only common tests should be described solely by name; describe more complex techniques in the Methods section.*
☒	☐	A description of all covariates tested
☒	☐	A description of any assumptions or corrections, such as tests of normality and adjustment for multiple comparisons
☐	☒	A full description of the statistical parameters including central tendency (e.g. means) or other basic estimates (e.g. regression coefficient) AND variation (e.g. standard deviation) or associated estimates of uncertainty (e.g. confidence intervals)
☐	☒	For null hypothesis testing, the test statistic (e.g. *F*, *t*, *r*) with confidence intervals, effect sizes, degrees of freedom and *P* value noted<br>*Give P values as exact values whenever suitable.*
☒	☐	For Bayesian analysis, information on the choice of priors and Markov chain Monte Carlo settings
☒	☐	For hierarchical and complex designs, identification of the appropriate level for tests and full reporting of outcomes
☒	☐	Estimates of effect sizes (e.g. Cohen's *d*, Pearson's *r*), indicating how they were calculated

Our web collection on statistics for biologists contains articles on many of the points above.

Software and code

Policy information about availability of computer code

Data collection	No software was used for data collection
Data analysis	For the transcriptomic-based<br>FastQC (v.0.11.9)<br>fastp (v.0.23.0)<br>Trimmomatic (v.0.39)<br>STAR (v.2.7.4a)<br>DESeq2 (v.1.26.0)<br>topGO (v. 2.38.1)<br>ggpubr (v.0.4.0)<br>ggplot2 (v.3.3.6)<br>WGCNA (v.1.71)<br>For parentage assignment and other experiment data analyses, the following software tools were used:<br>R v4.1.1 , SPSS v.29.0, t-tests, Fisher's test or G-tests.<br>Here is the public link for the Github repository: https://github.com/Joscolgan/bombus_mated_worker_analysis

For manuscripts utilizing custom algorithms or software that are central to the research but not yet described in published literature, software must be made available to editors and reviewers. We strongly encourage code deposition in a community repository (e.g. GitHub). See the Nature Portfolio guidelines for submitting code & software for further information.

Data

Policy information about availability of data

All manuscripts must include a data availability statement. This statement should provide the following information, where applicable:

- Accession codes, unique identifiers, or web links for publicly available datasets
- A description of any restrictions on data availability
- For clinical datasets or third party data, please ensure that the statement adheres to our policy

All RNA-seq datasets used in the present study have been deposited in the Sequence Read Archive database hosted by the National Center for Biotechnology Information (BioProject Accession ID: PRJNA868857). Additional datasets, such as gene-level read count matrices, are provided as supplemental files to assist in the reanalysis of the transcriptomic-based analyses.

In terms of other resources used, we used the publicly available Bombus terrestris reference genome assembly (Bter_1.0; Ensembl Metazoa), the information for which is listed, including appropriate citation of associated manuscript, in the Methods section of our manuscript.

Human research participants

Policy information about studies involving human research participants and Sex and Gender in Research.

Reporting on sex and gender	Not applicable
Population characteristics	Not applicable
Recruitment	Not applicable
Ethics oversight	Not applicable

Note that full information on the approval of the study protocol must also be provided in the manuscript.

Field-specific reporting

Please select the one below that is the best fit for your research. If you are not sure, read the appropriate sections before making your selection.

☐ Life sciences ☐ Behavioural & social sciences ☒ Ecological, evolutionary & environmental sciences

For a reference copy of the document with all sections, see nature.com/documents/nr-reporting-summary-flat.pdf

Ecological, evolutionary & environmental sciences study design

All studies must disclose on these points even when the disclosure is negative.

Study description	We examined whether worker spermathecae are functional through artificial insemination experiments and compared their gene expression post-insemination of workers and queens. We also tested whether inseminated workers can rear colonies and produce female offspring throughout the colony life-cycle. We further tested whether the mating of workers is influenced by social factors post-hatching. Lastly, using semi-field experiments, we tested whether workers can naturally mate in de-queened colonies and go on to produce queen offspring.
Research sample	For the bumblebee worker-based mating and colony-founding experiments, we used at least 30 samples per experimental group and repeated trials at least three times. To determine whether artificially inseminated workers and queens undergo similar changes in gene expression, we conducted targeted transcriptomic analyses of the reproductive organs (spermatheca, vagina, and median oviduct), as well as the brains, fat bodies, and ovaries of artificially inseminated and control workers and queens. Our total RNA-seq dataset consisted of 256 samples.
Sampling strategy	We collected wild queens of Bombus lantschouensis and Bombus ignitus from Gansu province (E: 106.10, N: 34.26), China in April and May 2019, and reared them to produce colonies under laboratory conditions in environmentally-controlled rooms (temperature 28°C±1°C, relative humidity 60% ± 5%, in the dark). For Bombus montivagus, workers and males were collected from the field (E:103.89, N:24.78). Commercial colonies of Bombus terrestris were obtained from a commercial supplier based at the Institute of Apicultural Research, Chinese Academy of Agricultural Sciences.
Data collection	RNA-seq data generation was performed on an Illumina Novaseq 6000 by OE Biotech Co., Ltd. (Shanghai, China).
Timing and spatial scale	Data collection occurred between 2019 and 2022. In brief, field sampling was performed in 2019 while laboratory-based experiments, including the generation of samples for RNA-seq, were performed between 2019 and 2022.
Data exclusions	For RNA-seq comparisons, certain samples were removed from each tissue based on aberrant gene expression profiles identified through principal component and hierarchical-based clustering analyses.
Reproducibility	We have provided gene-level count matrices, as well as scripts, for the reanalysis of the transcriptomic datasets.

Randomization	Bees were randomly assigned to different treatment groups.
Blinding	Experimenters were not blinded to group allocation, which is not practical as different groups needed specific treatments (e.g. with or without bees administraction). All data analyses were performed automatically with the same parameter setting for each experiment.

Did the study involve field work? ☐ Yes ☒ No

Reporting for specific materials, systems and methods

We require information from authors about some types of materials, experimental systems and methods used in many studies. Here, indicate whether each material, system or method listed is relevant to your study. If you are not sure if a list item applies to your research, read the appropriate section before selecting a response.

Materials & experimental systems

n/a	Involved in the study	
☒	☐	Antibodies
☒	☐	Eukaryotic cell lines
☒	☐	Palaeontology and archaeology
☐	☒	Animals and other organisms
☒	☐	Clinical data
☒	☐	Dual use research of concern

Methods

n/a	Involved in the study	
☒	☐	ChIP-seq
☒	☐	Flow cytometry
☒	☐	MRI-based neuroimaging

Animals and other research organisms

Policy information about studies involving animals; ARRIVE guidelines recommended for reporting animal research, and Sex and Gender in Research

Laboratory animals	Commercial colonies of Bombus terrestris were obtained from a commercial supplier based at the Institute of Apicultural Research,Chinese Academy of Agricultural Sciences.
Wild animals	Workers of the following species were collected from local sites for the purpose of producing female offspring and mating trials: Oueen of Bombus lantschouensis and Bombus ignitus, and workers of Bombus montivagus collected from field using insect net and kept them in cages.
Reporting on sex	Our study examined the mating potential of bumblebee workers, which are all female. Given the primary aim of our study was to understand if they can mate and produce colonies, the majority of our analyses are female-based.
Field-collected samples	We collected wild queens of 161 Bombus lantschouensis and 100 Bombus ignitus from Gansu province (E: 106.10, N: N: 34.26), China in April and May 2019, and reared them to produce colonies under laboratory conditions in environmentally-controlled rooms (temperature 28°C±1°C, relative humidity 60% ± 5%, inin the dark). For one Bombus montivagus group, workers and males were collected from the field (E:103.89, N:24.78).
Ethics oversight	Note that full information on the approval of the study protocol must also be provided in the manuscript.

Note that full information on the approval of the study protocol must also be provided in the manuscript.
